# Effectiveness of behavioural tobacco cessation interventions with and without pharmacotherapy among people living with HIV in Viet Nam: a three-arm pragmatic randomised controlled trial

**DOI:** 10.1016/S2214-109X(25)00451-6

**Published:** 2026-03

**Authors:** Donna Shelley, Mari Armstrong-Hough, Trang Nguyen, Gloria Guevara Alvarez, Reet Kapur, Jonathan Shuter, Lloyd Goldsamt, Yesim Tozan, Hoang Van Minh, Giap Van Vu, Phuong Thu Phan, Charles M Cleland, Nam Nguyen

**Affiliations:** School of Global Public Health (Prof D Shelley MD, M Armstrong-Hough PhD, Y Tozan PhD, G Guevara Alvarez PhD, R Kapur MPH), Grossman School of Medicine (C M Cleland PhD), and Rory Meyers College of Nursing (L Goldsamt PhD), New York University, New York, NY, USA; Institute of Social and Medical Studies, Hanoi, Viet Nam (N Nguyen DrPH, T Nguyen MA); Albert Einstein College of Medicine, New York, NY, USA (Prof J Shuter MD); Hanoi University of Public Health, Hanoi, Viet Nam (Prof H Van Minh PhD); Bach Mai Hospital, Hanoi, Viet Nam (G Van Vu PhD); Hanoi Medical University, Hanoi, Viet Nam (P Thu Phan MD)

## Abstract

**Background:**

People living with HIV are two to three times more likely to smoke than the general population, resulting in higher risk for tobacco-related morbidity and mortality. Despite this growing burden of disease, there is little evidence for the long-term effectiveness of tobacco cessation interventions among people living with HIV, particularly in low-income and middle-income countries. We aimed to compare the effectiveness of three tobacco cessation interventions among people living with HIV.

**Methods:**

We conducted an open-label, three-arm pragmatic randomised controlled trial in 13 outpatient HIV clinics (OPCs) in Hanoi, Viet Nam. Adults who smoked at least one cigarette a day, lived in Hanoi, had a clinic visit in the past 12 months, and had daily access to a mobile telephone that could receive text messages were allocated (1:1:1) to either: proactive referral to Viet Nam’s national smokers’ Quitline counselling programme (Quitline group); six-session tailored counselling delivered by trained OPC nurses plus text messages (Counselling + SMS group); or Counselling + SMS plus 6 weeks of nicotine replacement therapy (ie, 2 mg nicotine gum; Counselling + SMS+ gum group). Randomisation was by stratified permuted block randomisation with block sizes of three and six. Neither study participants, OPC health-care workers, nor study staff were masked to group assignment. All patients received advice to quit and brief cessation counselling during their physician visit. The primary outcome was 7-day point-prevalence smoking abstinence confirmed at 6 months by exhaled carbon monoxide concentration of less than 8 ppm, assessed with an intention-to-treat analysis. The trial was registered on Dec 17, 2021, at ClinicalTrials.gov (NCT05162911).

**Findings:**

Between Nov 30, 2021 and Sept 27, 2023, 672 patients were randomly allocated to the three test groups (221 to the Quitline group, 225 to the Counselling + SMS group, and 226 to the Counselling + SMS + gum group). 338 (50%) patients reported dual waterpipe and cigarette use. At 6 months, 109 (16%) patients had confirmed abstinence (28 [13%] for Quitline, 40 [18%] for Counselling + SMS, and 41 [18%] for Counselling + SMS + gum). There were no significant differences between intervention groups: Counselling + SMS versus Quitline (odds ratio 1·48, 95% CI 0·78–2·81; p=0·33), Counselling + SMS+ gum versus Quitline (1·64, 0·86–3·11; p=0·17), and Counselling + SMS + gum versus Counselling +SMS (1·11, 0·61–2·00; p=0·91). There were no serious adverse events linked to the study interventions throughout the trial duration.

**Interpretation:**

Integrating nurse-delivered cessation treatment and proactive referral to a national Quitline was feasible within the context of HIV care. In the absence of evidence that tailored interventions provide additional benefit, our findings suggest that national Quitlines, available in 42 low-income and middle-income countries, might serve as a resource for integrating tobacco treatment into HIV care systems.

**Funding:**

US National Cancer Institute.

## Introduction

People living with HIV are two to three times more likely to smoke and have comparatively less success in achieving abstinence.^1,2^ This modifiable risk factor contributes to substantial AIDS-related and non-AIDS-related morbidity and mortality in this group.^3^ As a result, people living with HIV who are receiving antiretroviral therapy (ART) now lose more life-years due to smoking than due to HIV infection.^3,4^ The substantial tobacco-related adverse health effects threaten to undermine the effectiveness of global HIV/AIDS programmes, particularly in low-income and middle-income countries (LMICs) where more than 80% of tobacco users reside.^5,6^

Substantial evidence exists on the effectiveness of a range of pharmacotherapies and behavioural interventions for people in the general population who use tobacco.^7–9^ In contrast, systematic reviews have found insufficient evidence that interventions that are efficacious among the general population yield similar outcomes among people living with HIV.^1,10,11^ Although people living with HIV who use tobacco are interested in quitting, they experience a range of sociobehavioural and medical challenges that might impede smoking abstinence.^12^ These challenges include high rates of comorbidities, such as depression, social isolation, and substance use, which are associated with lower rates of cessation.^12,13^

The only behavioural intervention that has demonstrated long-term efficacy among people living with HIV is Positively Smoke Free, an eight-session intervention, grounded in social cognitive theory and tailored to the needs of people living with HIV.^14^ A trial^15^ conducted in Kenya found that a culturally adapted version of the Positively Smoke Free programme and bupropion were each effective in promoting 6-month abstinence compared with control conditions. The combination was associated with higher 6-month abstinence rates than either therapy alone. Findings from this trial suggest that tobacco cessation interventions might need to be tailored to the needs of people living with HIV, adapted to local context, and offer more intensive multisession counselling with or without pharmacotherapy to achieve long-term smoking cessation among people living with HIV.

System-level interventions that facilitate tobacco cessation treatment in the context of HIV care are also understudied.^11^ These interventions include policies and practices that support routine screening for tobacco use, brief clinician advice, and connecting patients to tobacco use treatment, either onsite or through a referral to external resources such as telephone counselling (eg, national Quitlines).^9^ The WHO guidance for integrating non-communicable diseases in HIV/AIDS programmes calls for systemwide integration of tobacco cessation practices.^9,16^ Integrating treatment at the point of care has the potential to increase patient access to counselling and treatment, regardless of their readiness to quit. Research is needed to identify both cost-effective cessation interventions and system-level strategies for implementing tobacco use treatment in HIV care settings, particularly in LMICs.

Viet Nam has one of the highest smoking prevalence rates in the world.^17^ Among the general population, 41% of men use tobacco. Waterpipe use is the next most common form of tobacco use (14%), and dual waterpipe and cigarette use are common. In a previous study,^18^ more than 35% of men reported using both tobacco products. Among people living with HIV, smoking prevalence is more than 50%. However, data on poly tobacco use in this population and its effect on tobacco cessation outcomes are insufficient.

To address gaps in the literature, particularly the small number of studies conducted in LMICs, in collaboration with the Viet Nam Administration for HIV/AIDS Control and Viet Nam’s National Quitline, we conducted a pragmatic randomised controlled trial to compare the effectiveness of three tobacco cessation interventions among people living with HIV receiving care in outpatient HIV clinics (OPCs) in Hanoi, Viet Nam. Our trial tested the hypothesis that OPC-based, nurse-delivered multisession counselling tailored to people living with HIV, would result in higher abstinence rates than counselling delivered by Viet Nam’s national Quitline, a sustainable, free resource, designed for the general population of people who use tobacco. We also hypothesised that adding nicotine replacement therapy (NRT) to tailored counselling would increase abstinence compared with the Quitline and tailored counselling alone.

## Methods

### Study design

We conducted an open-label pragmatic randomised controlled trial, in which participants were randomly assigned to one of three treatment groups: proactive referral to Viet Nam’s National Smokers’ Quitline counselling programme (Quitline group); six-session counselling tailored to the needs of people living with HIV living in Viet Nam and delivered by trained OPC nurses plus text messages (Counselling + SMS group); or Counselling plus SMS plus 6 weeks of NRT (ie, 2 mg nicotine gum [Viet Phuc Pharma, Can Tho City, Viet Nam]; Counselling + SMS + gum). A proactive approach to link patients to the national Quitline was selected because it is associated with higher rates of connectivity compared with a reactive approach, in which patients are given the number to call.^19^ The study was conducted in 13 OPCs located in Hanoi. Sites were eligible if they had a minimum of 240 active patients and four health-care providers, including one medical director. Site recruitment procedures are detailed in a previous publication.^19^ The study was approved by the New York University Langone Health Office of Science and Research Institutional Review Board (approval number: il9-01783) and the Institutional Review Board in Biomedical Research, Institute of Social and Medical Studies (approval number: 00007993) and is registered with ClinicalTrials.gov (NCT05162911).

### Participants

All participants were receiving care in one of the 13 OPCs. Patients were included if they were aged 18 years and older, smoked at least one cigarette every day, lived in Hanoi, had a clinic visit in the past 12 months, and had daily access to a mobile telephone that could receive text messages. Patients were excluded if they were currently using tobacco cessation medication, enrolled in a smoking cessation programme (eg, National Quitline), reported a contraindication to using nicotine gum, or were pregnant or breastfeeding.

During the study period, all patients were screened for cigarette use by the nurse who registers patients for their routine visit. Nurses used a standard form that included the question, “Do you now smoke cigarettes every day, some days, or not at all?” Patients responding every day were, with their permission, referred to the onsite research assistant to receive additional information about the study purpose and procedures. Patients willing to continue the enrolment process provided written consent and completed the baseline survey. For patients randomly assigned to receive Quitline counselling, the consent process also included a request for permission to share contact information with the Quitline. Once enrolled and randomly assigned, patients continued with their physician visit, during which all patients received advice to quit and brief cessation counselling. This process was standard care across all three study conditions. Patients who declined to participate were offered a smoking cessation brochure with the Viet Nam National Quitline telephone number. Participants received US$3 for each survey completed.

### Randomisation and masking

Patients who met the eligibility criteria, provided written consent, and completed the baseline survey were enrolled and randomly assigned to one of three study conditions in a 1:1:1 ratio, with the use of stratified permuted block randomisation with block sizes of three and six. Stratification was done by OPCs. The study statistician generated the allocation table, which was concealed using the REDCap randomisation module.^21^ This method allowed the research assistant, who was conducting onsite enrolment, to request an assignment for an individual participant. The project manager in Viet Nam had access to the allocation table to allow group assignments when internet access to REDCap (version 13.8.1) was temporarily unavailable. Consistent with the pragmatic design, once an assignment to study condition was made, study participants, OPC health-care workers, and study staff were not blinded to group assignment.

### Procedures

Before launching the trial, formative research, conducted in collaboration with the Viet Nam Administration for HIV/AIDS Control, the National Quitline, District Health Directors and OPC medical directors, nurses, and patients, guided the process of adapting and tailoring the intervention components for people living with HIV in Viet Nam and informed changes to clinic systems (eg, tobacco use screening and Quitline referral systems).^19^

Participants assigned to the Quitline group received information about the Quitline service from a trained OPC nurse after they completed their routine physician visit. Participant information was sent to the Quitline via a secure web-based system. Once this information was received, the Viet Nam Quitline initiated their standard counselling protocol. This process included a maximum of ten counselling sessions for up to 12 months, five in the first month of enrolment and the remainder between the second and 12th month. Quitline counsellors use a standardised manual to guide the counselling sessions. The manual focuses on enhancing motivation, developing coping strategies, and increasing confidence in executing a quit plan.^22^ Quitline counsellors are nurses or public health professionals employed full-time by Bach Mai Hospital’s National Quitline. Bach Mai provides counsellor training and ongoing supervision.

Participants assigned to the Counselling + SMS groups received six in-person counselling sessions for up to 4·5 months. OPC nurses, whose role is to provide ART adherence counselling and dispense medications, delivered the multisession counselling. Once randomly assigned, participants were referred to the onsite nurse counsellor to schedule the initial session. The five follow-up sessions occurred in weeks 2, 3, 4, 8, and 12 and lasted approximately 1 h. Nurses used a manual to guide each session. The manual was adapted from previous tobacco cessation research conducted in Viet Nam and incorporated principles of motivational interviewing, social cognitive theory, and relevant elements of Positively Smoke Free that were tailored to this population with the use of salient sociocultural and behavioural concepts that emerged from our formative research.^15,20,23^

Participants assigned to the Counselling + SMS + gum group were given a 6-week supply of 2 mg nicotine gum in intervals that coincided with scheduled counselling sessions. The gum was dispensed by the clinic pharmacist. Participants in the Counselling+SMS and Counselling + SMS + gum groups also received two text messages per day for 8 weeks and then daily for 4 weeks. The SMS library was adapted from the WHO health programme that was previously translated and culturally tailored to a general population of people who use tobacco in Viet Nam.^18^ Messages were designed to supplement counselling by further enhancing knowledge about the harms of tobacco use and motivation to quit, change outcome expectancies (eg, benefits of quitting), and offer cognitive and behavioural strategies such as refusal skills.^20^

Several strategies were used to enhance feasibility and implementation fidelity. All OPC health-care workers, including pharmacists, attended 1·5 days of skills-based training to increase their capacity to screen for tobacco use, offer advice to quit, and provide brief counselling.^9,24^ hysicians were given a one-page coaching guide to support consistent delivery of advice and brief cessation counselling, which included relevant questions and probes.^20^ OPC nurses who delivered the multisession counselling intervention received separate 3-day training. This training included reviewing the content from the health-care worker training plus training on the use of nicotine gum, motivational interviewing techniques to support tobacco cessation, role-playing each of the six counselling sessions, and training on how to use the counselling tools, which included the manual, worksheets, and tracking forms. Nurses attended 1-day booster training 6 months after the initial training. After the initial training, counsellors participated in monthly supportive supervision meetings with trained research staff.^20^ These meetings included case reviews and structured feedback informed by systematic audits of randomly audiotaped sessions.

### Outcomes

The primary outcome was 6-month carbon monoxide-confirmed, 7-day point prevalence abstinence among all randomly assigned patients. For dual users, abstinence was defined as carbon monoxide-confirmed abstinence from both cigarettes and waterpipe.^25^ Trained research staff assessed self-reported 7-day smoking abstinence by asking the following question, “In the past 7 days have you smoked even a puff of a cigarette?”^26,27^ The same question was used to assess waterpipe abstinence. Carbon monoxide tests were conducted to confirm self-reported smoking abstinence. For dual users, carbon monoxide testing was conducted if participants reported 7-day abstinence from both waterpipe and cigarettes. Carbon monoxide was measured with the use of the piCO+ Smokerlyzer (coVita, Haddonfield, NJ, USA), which was maintained and calibrated according to manufacturer’s recommendations. We used the recommended exhaled carbon monoxide cutoff of less than 8 ppm.^28^

Sociodemographic information (ie, age, sex, education, and household income level), smoking behaviours (type of and frequency of tobacco use per day),^26,27^ alcohol use (alcohol use disorders identification test-consumption [AUDIT-C)]),^29^ any drug use in the past three months, depression symptoms (Center for Epidemiologic Studies Depression Scale [CES-D 8]),^30^ overall health status,^31^ and the Fagerström test of nicotine dependence (FTND; range 1–10: 0–2 very low dependence, 3–4 low, 5 moderate, 6–7 high, and 8–10 very high) were assessed at baseline.^32^ Data on sex were self-reported, with participants choosing from options male or female. Data on gender were not collected. All patient survey data were collected and managed with the use of REDCap electronic data capture tools hosted at New York University, NY, USA.^21^

Intervention fidelity was assessed for each intervention component. Both Quitline and OPC nurse counsellors used REDCap to document all completed sessions (ie, date, telephone session number, and duration) and used checklists, which aligned with the counselling manual content, to document the content discussed.^21^ OPC pharmacists were responsible for dispensing nicotine gum and used a standardised form to document the date, time, and number of boxes of nicotine gum patients received. Receipt of, and interaction with text messages, was assessed with the use of the 3-month end-of-treatment participant survey.

A Data Safety and Monitoring Board met twice per year to assess any adverse events. A standardised form was used to document any safety incidents, which captured the participant’s ID, date and type of event, and its relation to the study intervention.

### Statistical analysis

Sample size was determined by simulating data from 13 OPC sites with a modest prevalence of carbon monoxide-confirmed abstinence in the Quitline group (0·10), and increased abstinence in the Counselling + SMS (0·20) and Counselling + SMS + gum (0·32) groups. In conditional logistic regression analysis of 10 000 simulated datasets, we found that all pairwise group differences could be detected with at least 80% power if the sample size was 672 (224 per group; appendix p 1). The primary comparative analysis included all participants as randomly assigned (ie, intention-to-treat) and used conditional logistic regression to compare study groups with carbon monoxide-confirmed tobacco abstinence at 6 months as the primary outcome. Clinic was included in the model as a fixed effect. The conditional logistic regression analysis focuses on pooled within-clinic differences in outcome. With this approach, variation in outcomes due to between-clinic differences is separated out by means of the clinic fixed effects. Type of tobacco use at baseline (cigarette only *vs* dual use of cigarettes and water pipe) and the FTND score were included as covariates. The literature, and previous research in Viet Nam, demonstrate that both variables are significant predictors of tobacco cessation.^33^ The analysis was adjusted for multiple pairwise comparisons of study group with the use of Tukey’s method. For participants without a 6-month follow-up and individuals who reported abstinence at the 6-month follow-up but were missing carbon monoxide confirmation, continued smoking was assumed. To analyse nicotine dependence among dual users, we separately calculated dependence scores for cigarettes and waterpipes and chose the higher of the two to represent overall nicotine dependence.^34^ In addition to intention-to-treat analysis, analysis of patients with no missing data (ie, complete cases) was performed to determine the potential effect of assuming continued smoking for patients missing the primary outcome. Descriptive statistics were used to summarise baseline characteristics. Continuous variables were presented as means with SDs and ranges, whereas categorical variables were summarised as frequencies with percentages. All significance testing was two-tailed, and p<0·05 indicated statistical significance. All statistical analyses were performed with the use of R software (version 4.4.2).

### Role of the funding source

The funder of the study had no role in study design, data collection, data analysis, data interpretation, or writing of the report.

## Results

Between Nov 30, 2021 and Sept 27, 2023, we enrolled 672 adults who were receiving ART in 13 OPCs in Hanoi, Viet Nam. Initial screening yielded 1678 patients who met the criteria as a current smoker (figure). Among these patients, 828 declined to receive further information about the study (figure). The final study cohort consisted of 672 participants randomly allocated to the three study conditions. There were no serious adverse events linked to the study interventions throughout the trial duration.

Table 1 shows the baseline demographic and clinical characteristics of the study population by intervention group. The mean age of participants was 44·4 years (SD 7·1, range 19–71). Consistent with low smoking prevalence among women in Viet Nam (<1%),^17^ 647 (96%) patients were men. Less than half of participants reported a primary or secondary level of education (n=294, 44%). Most participants belonged to households earning less than 300 million Vietnamese Dong (~US$12 000) annually (n=589, 88%) and about half of the participants were married (n=366, 55%). The majority reported fair-to-poor health status (n=469, 70%); more than a third met the criteria for clinically significant symptoms of depression (score of ≥9 on the CES-D 8 scale [n=250, 37%]), 386 (57%) met the criteria for hazardous drinking, and 109 (16%) reported drug use in the past 3 months. The mean FTND score was 4·9 (SD 2·4). The mean number of cigarettes used per day was 14·5 (SD 8·2) and the mean number of times per day participants reported waterpipe use was 10·1 (9·9). 338 (50%) participants reported dual use of waterpipe and cigarettes. Participants who used cigarettes only, or were dual users, were distributed evenly across intervention groups.

93 patients (14%) did not complete a 6-month follow-up. An additional 36 patients (5%) reported abstinence in the 6-month follow-up survey but did not complete carbon monoxide confirmation. In the intention-to-treat analysis, continued smoking was assumed for these 129 patients. There were no significant differences in demographic characteristics, mental health, substance use, and nicotine dependence between individuals with and without the primary outcome fully observed (data not shown).

Table 2 shows 7-day point-prevalence smoking abstinence with the use of intention-to treat analysis by intervention conditions. At 6 months, 109 patients had confirmed abstinence (16%). There was no significant difference between intervention conditions for the total sample. Differences between the Counselling + SMS and Quitline groups (odds ratio=1·48; 95% CI 0·78–2·81; p=0·33), the Counselling + SMS + gum and Quitline groups (1·64; 0·86–3·11; p=0·17), and the Counselling + SMS + gum and Counselling + SMS groups (1·11; 0·61–2·00; p=0·91) were not statistically significant, and data could not overcome the null hypotheses of no pairwise group differences (table 3). Similarly, abstinence rates did not differ significantly by intervention for cigarette-only or dual users (table 2). When the type of tobacco use was explored as a potential moderator of group differences, group differences appeared to be larger among dual users, but none of the pairwise simple main effects of group were statistically significant (data not shown).

Table 4 shows smoking abstinence rates with the use of the complete case analysis. This analysis yielded similar results with no group differences in abstinence among cigarette-only or dual users (table 4) or overall (table 5).

Among participants referred to the Quitline, 194 (88%) completed at least one telephone counselling session, 113 (51%) completed more than five sessions, and 33 (15%) completed all ten sessions. 402 (89%) patients randomly assigned to nurse-delivered counselling (Counselling + SMS and Counselling + SMS + gum) completed all six sessions, and 438 (97%) completed at least one session, with no differences in the results between the two counselling conditions (data not shown). Nurse counsellors achieved a high rate of fidelity to the manual content (90% of content reviewed at each session with no differences between the two counselling groups). 164 (73%) patients received the full dose of 42 boxes of nicotine gum. 393 (98%) in the Counselling + SMS and Counselling + SMS + gum groups received text messages during the trial period, and 317 (79%) of them reported that they usually or always read those messages. 31 patients (14%) in the Quitline group were still receiving treatment at the time that 6-month abstinence was assessed; 157 (71%) completed treatment within the same time as the tailored counselling group (ie, 4·5 months), and the mean length of treatment was the same across intervention groups.

## Discussion

We found no significant differences in 6-month smoking abstinence across the three intervention groups for the total sample. All three approaches achieved long-term cessation rates exceeding those previously reported.^11^ For comparison, a 2024 Cochrane systematic review^11^ reported average 6-month cessation rates among people living with HIV of 11% with behavioural support alone, and 14% with behavioural support plus NRT. All identified trials were conducted in high-income or middle-income countries. This review also found insufficient evidence to determine whether tailored tobacco cessation interventions are more effective than non-tailored approaches, as none included a non-tailored comparison group.^11^

To the best of our knowledge, this is the first randomised trial conducted in an LMIC to show that a multisession national Quitline programme, designed for a general population, can achieve smoking cessation outcomes that are similar to those of a tailored behavioural counselling intervention. There is strong evidence that Quitlines are cost-effective and increase the reach of tobacco cessation services.^19^ Our findings suggest that in the absence of evidence that tailored interventions provide additional benefit, national Quitlines, available in 42 LMICs, offer a referral resource and practical strategy for integrating tobacco use treatment within HIV services.

Lower quit rates among dual users can be explained by higher levels of nicotine dependence and recent drug use in this group (data not shown) compared with those who used cigarettes only.^36^ Both characteristics are associated with less success in quitting.^13,37^ Despite increases in poly use globally, we are not aware of previous trials that have evaluated the effectiveness of cessation interventions among people living with HIV who use tobacco products other than cigarettes. Research is needed to further define determinants of poly use and identify factors that influence cessation to inform the design of effective interventions for this growing population of tobacco users.

There is evidence that combining behavioural and pharmacotherapy interventions enhances smoking cessation compared with either approach alone among the general population of people who use cigarettes.^9^ However, consistent with previous trials among people living with HIV, combining NRT with tailored behavioural counselling was not associated with higher abstinence rates compared with tailored behavioural counselling alone. Some studies^15,38^ suggest that for people living with HIV, varenicline and bupropion might result in higher rates of cessation compared with control conditions. Yet, in many LMICs, cost is a barrier to accessing pharmacotherapy. For example, Viet Nam does not offer cessation medication through national insurance. Our findings suggest that if pharmacotherapy is not available, affordable, or acceptable to the patient, behavioural interventions, including referral to a Quitline or other population-based cessation intervention alone might improve cessation outcomes and should be offered to people living with HIV.^9^

The trial adds to the literature by demonstrating the feasibility of integrating routine screening for tobacco use, brief counselling, and a range of behavioural interventions, in the context of HIV care in an LMIC. Several strategies were used to enhance feasibility and increase fidelity to intervention protocols. These strategies included changes in the chart system (ie, documentation of tobacco use, referral systems), clinician training, supportive supervision, and clinical tools (eg, brief counselling guide) that were adapted in collaboration with OPC health-care workers.^20^ These approaches align with global guidance for integrating non-communicable disease prevention and treatment programmes in the context of HIV care.^16^

LMICs are exploring a range of service models to achieve this goal that include facilitating referrals through partnerships with institutions or health systems (eg, tuberculosis or primary care clinics) that offer cessation services.^16^ Screening all patients for tobacco use, offering brief counselling, and a proactive referral to existing cessation resources (also known as Ask, Advise, Connect), has the potential to broaden the reach and effect of existing cessation services.^9,12^ Moreover, enabling physicians to delegate the time-consuming task of multisession counselling to external resources, such as Quitlines, might enhance both the feasibility and long-term sustainability of integrating tobacco use treatment into routine HIV care. Additional data are needed to inform policy and funding decisions related to designing and evaluating effective models for scaling tobacco use treatment across HIV care systems in LMICs.

Our study has several limitations. First, the finding of no significant group differences is not evidence of equivalence; rather, the study might have been underpowered to detect smaller but clinically relevant differences. Further, the absence of significant differences between the Quitline and tailored counselling might have been explained by the greater number of sessions offered (ten sessions *vs* six) and potential for a longer treatment period (up to 12 months). However, the majority of participants in the Quitline group (71%) did complete treatment within the same timeframe as that of the tailored counselling groups. Additionally, the SMS component of tailored treatment offered additional opportunities for support that might be expected to compensate for fewer sessions compared with the Quitline protocol. Second, we were unable to verify consistent use of the nicotine gum, which might have influenced treatment outcomes. In addition, combining a short-acting and long-acting nicotine medication is more effective than administering either alone.^8^ However, resource constraints precluded prescribing more than one NRT product. Finally, nearly half of eligible patients declined participation. Individuals enrolled might have been more motivated to quit. In practice, opt out approaches that refer patients to treatment unless they actively decline can increase patient engagement and warrant further study in HIV care settings.

Integrating screening, brief counselling, and system changes that facilitate connecting patients to onsite multisession tailored counselling or national Quitline counselling, was feasible within the context of HIV care. Our findings extend the current literature by demonstrating that a multisession national Quitline programme designed for the general population of people who use tobacco can achieve cessation outcomes similar to those of a tailored behavioural intervention. Leveraging existing Quitline infrastructure could be an efficient strategy for increasing access to tobacco use treatment within HIV care systems.

## Supplementary Material

1

## Figures and Tables

**Figure:**
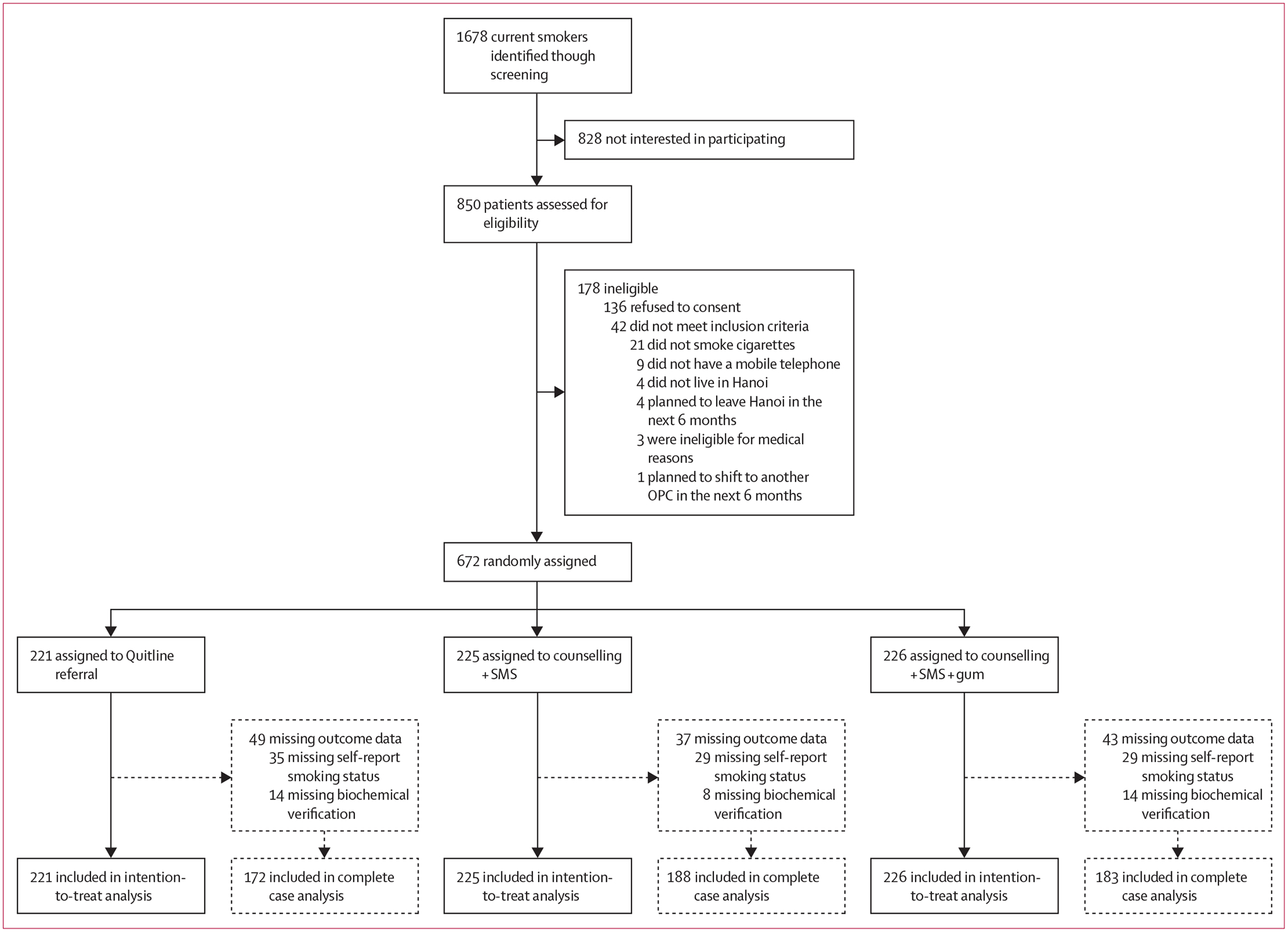
Trial profile OPC=outpatient HIV clinic.

**Table 1:** Description of study population by intervention group

	Quitline referral (n=221)	Counselling+SMS (n=225)	Counselling+SMS+gum (n=226)	Total (n=672)
Age, years	43·8 (7·3)	44·4 (7·4)	45·0 (6·8)	44·4 (7·1)
Men	212 (96%)	217 (96%)	218 (97%)	647 (96%)
Women	9 (4%)	8 (4%)	8 (3%)	25 (4%)
Years living with HIV	12·6 (6·3)	11·7 (6·2)	12·6 (6·4)	12·31 (6·3)
Education				
Less than high school	96 (44%)	108 (48%)	90 (40%)	294 (44%)
High school (grade 10–12)	80 (36%)	80 (36%)	94 (42%)	254 (38%)
Vocational training, college, university, or graduate degree	45 (20%)	37 (16%)	42 (18%)	124 (18%)
Household income <300 million Vietnamise dong (~US$12 000) in the past 12 months*	192 (88%)	200 (91%)	197 (88%)	589 (88%)
Married	117 (53%)	120 (53%)	129 (57%)	366 (55%)
Fair-to-poor self-reported health status	145 (66%)	166 (74%)	158 (70%)	469 (70%)
Cigarette-only users	111 (50%)	117 (52%)	106 (47%)	334 (50%)
Dual cigarette and waterpipe users	110 (50%)	108 (48%)	120 (53%)	338 (50%)
Number of cigarettes used per day	14·6 (8·2)	13·6 (7·9)	15·4 (8·6)	14·5 (8·2)
Number of times per day waterpipe used†	10·5 (9·8)	9·30 (8·3)	10·4 (11·5)	10·1 (9·9)
Nicotine dependence score, (FTND: 1–10)	4·9 (2·3)	4·6 (2·5)	5·1 (2·5)	4·9 (2·4)
Depression symptoms positive screen, (CES-D 8, ≥9)	82 (37%)	90 (40%)	78 (35%)	250 (37%)
Hazardous drinking, n (%), (AUDIT-C ≥3 for women, AUDIT-C ≥4 for men)	137 (62%)	121 (54%)	128 (57%)	386 (57%)
Drug use in the last 3 months	40 (18%)	30 (13%)	39 (17%)	109 (16%)

Data are mean (SD) or number (%). AUDIT-C=alcohol use disorders identification test-consumption. CES-D 8=Center for Epidemiologic Studies Depression Scale. FTND=Fagerström test of nicotine dependence.

* The sample for household income is 662 because ten participants had missing data.

† The sample for number of times per day a waterpipe was used is 338 because only dual cigarette and waterpipe users reported using a waterpipe; the remaining 334 participants used cigarettes only.

**Table 2:** Biochemically confirmed (carbon monoxide < 8 ppm), 7-day point-prevalence abstinence by intervention group and type of tobacco use at 6-month follow-up with the use of intention-to-treat analysis

	Quitline referral (n=221)	Counselling + SMS (n=225)	Counselling + SMS + gum (n=226)	Overall (n=672)*
Confirmed abstinence of all users	28 (13%)	40 (18%)	41 (18%)	109 (16%)
Confirmed abstinence of cigarette-only users	18 (16%) of 111	24 (21%) of 117	23 (22%) of 106	65 (19%) of 334
Confirmed abstinence of dual users	10 (9%) of 110	16 (15%) of 108	18 (15%) of 120	44 (13%) of 338

* Intention-to-treat analysis includes all randomly assigned participants. Participants with missing outcome data (n=129; 93 with no 6-month follow-up, 36 with no carbon monoxide confirmation) were classified as not abstinent.

**Table 3:** Pairwise comparisons of 6-month abstinence in intention-to-treat analysis (n=672)

	OR (95% CI)	p value
Counselling+SMS vs Quitline	1·48 (0·78–2·81)	0·33
Counselling+SMS+gum vs Quitline	1·64 (0·86–3·11)	0·17
Counselling+SMS+gum *vs* C+SMS	1·11 (0·61–2·00)	0·91

OR=odds ratio.

**Table 4:** Biochemically confirmed (carbon monoxide < 8 ppm), 7-day point-prevalence abstinence by intervention group and type of tobacco use at 6-month follow-up with the use of complete case analysis

	Quitline referral (n=172)	Counselling + SMS (n=188)	Counselling + SMS+ gum (n=183)	Overall (n=543)*
Confirmed abstinence of all users	28 (16%)	40 (21%)	41 (22%)	109 (20%)
Confirmed abstinence of cigarette-only users	18 (21%) of 87	24 (24%) of 100	23 (28%) of 83	65 (24%) of 270
Confirmed abstinence of dual users	10 (12%) of 85	16 (18%) of 88	18 (18%) of 100	44 (16%) of 273

* Complete case analysis includes participants who completed a 6-month follow-up survey and, if reporting abstinence, completed carbon monoxide testing. Participants with missing smoking status or missing carbon monoxide testing were excluded.

**Table 5:** Pairwise comparisons of 6-month abstinence in complete case analysis (n=543)

	OR (95% CI)	p value
Counselling+SMS *vs* Quitline	1·38 (0·71–2·67)	0·49
Counselling+SMS+gum *vs* Quitline	1·56 (0·81–3·03)	0·26
Counselling+SMS+gum *vs* C+SMS	1·13 (0·62–2·09)	0·88

OR=odds ratio.

## Data Availability

This study’s data include sensitive patient information, such as HIV status, and cannot be publicly shared due to ethical and legal reasons. De-identified R datasets, an Excel data dictionary, and relevant study documents, including the study protocol and statistical analysis plan, are available upon reasonable request. Researchers can submit data requests to DS (donna.shelley@nyu.edu) or NN (ntnam@isms.org.vn) with a brief proposal outlining the intended use.

## Research in context

### Evidence before this study

We searched PubMed, EBSCO, and the Cochrane Library for peer-reviewed articles published in English from Jan 1, 2010 to April 30, 2025, with the use of the following terms: (“smoking cessation” OR “tobacco use cessation” OR “nicotine replacement therapy” OR “pharmacotherapy” OR “behavioral counseling” OR “Quitline” OR “waterpipe” OR “polytobacco”) AND (“HIV” OR “people living with HIV” OR “PLWH” OR “PWH”) AND (“randomised controlled trial” OR “systematic review” OR “scoping review”) AND (“health system” OR “intervention” OR “system change”). There is a substantial body of evidence to support a range of pharmacotherapies and behavioural interventions for people in the general population who use tobacco. In contrast, there is little research focused on developing and testing smoking cessation interventions for people living with HIV, particularly in low-income and middle-income countries (LMICs). A 2024 Cochrane systematic review included 15 trials that evaluated individual-level and group-level interventions and two that evaluated system-change interventions for people living with HIV; none were conducted in LMICs. The authors found insufficient evidence that a range of behavioural and pharmacotherapy interventions, alone or in combination, produce long-term (≥6 months) smoking abstinence among people living with HIV. However, a 2025 randomised two-by-two factorial design trial, conducted in Kenya, found that Positively Smoke Free, an eight-session behavioural intervention tailored to the needs of people living with HIV, and treatment with bupropion, were each effective in promoting 6-month abstinence compared with control conditions. The combination of these two methods was associated with higher 6-month abstinence rates than either therapy alone. Although this trial suggests that a tailored intervention can be effective, the 2024 systematic review was unable to conclude that tailored behavioural interventions are more efficacious than non-tailored interventions, primarily due to the insufficient number of studies that included a standardised non-tailored comparison group. Similarly, whether the combination of behavioural interventions and pharmacotherapy improves cessation outcomes compared with either alone in this population is not well established.

### Added value of this study

We recruited a large sample of patients receiving care in HIV clinics in Hanoi, Viet Nam, to participate in a three-arm pragmatic randomised controlled trial (RCT) that compared the effectiveness of a nurse-delivered multisession counselling intervention plus text messaging with or without nicotine replacement therapy to proactive referral to the Viet Nam national telephone Quitline. The proportion of patients who achieved 6-month abstinence was high across all three groups relative to previous research, with a non-significant advantage for tailored behavioural intervention with or without nicotine gum compared with the national Quitline. To the best of our knowledge, this is the first RCT conducted in any of the LMICs to show that a multisession national Quitline programme, designed for a general population of people who use tobacco, might achieve 6-month smoking abstinence rates that are similar to those associated with a more resource-intensive nurse-delivered behavioural counselling intervention tailored to the specific needs of people living with HIV.

### Implications of all the available evidence

Efforts to implement tobacco use treatment in the context of HIV care are hindered by the scarcity of research focused on developing and testing interventions for people living with HIV, particularly in LMICs. Current evidence does not support supplementing behavioural interventions with nicotine replacement therapy. Other forms of pharmacotherapy, such as bupropion, might enhance cessation outcomes, but high costs and insufficient insurance coverage restrict access to these medications in LMICs. In the absence of interventions tailored to people living with HIV, national Quitlines, population-based interventions that are available in 42 LMICs, might serve as an effective resource for people living with HIV, particularly for those who use cigarettes only. However, waterpipe and cigarettes users might require a tailored intervention due to unique challenges to quitting. Integrating treatment at the point of care, including a proactive referral to existing cessation resources, has the potential to broaden the reach and effect of existing cessation services. Further research is needed to identify both cost-effective cessation interventions for people living with HIV and system-level strategies to optimise implementation of tobacco use treatment in HIV care settings in LMICs. More research on effective interventions for people who use more than one tobacco product is also needed.
