# Supplementary material for: Effectiveness of behavioural tobacco cessation interventions with and without pharmacotherapy among people living with HIV in Viet Nam: a three-arm pragmatic randomised controlled trial
Source: Lancet Glob Health. Author manuscript; Available in PMC 2026 Mar 2. (PMC12952184; doi:10.1016/S2214-109X(25)00451-6)
Supplement: 1 [file NIHMS2150007-supplement-1.pdf]

THE LANCET
Global Health

Supplementary appendix

This appendix formed part of the original submission and has been peer reviewed. We post it as supplied by the authors.

Supplement to: Shelley D, Armstrong-Hough M, Nguyen T, et al. Effectiveness of behavioural tobacco cessation interventions with and without pharmacotherapy among people living with HIV in Viet Nam: a three-arm pragmatic randomised controlled trial. *Lancet Glob Health* 2026; **14:** e407–16.

STATISTICAL ANALYSIS PLAN AND STUDY PROTOCOL

Trial title: Implementing tobacco use treatment in HIV clinics in Vietnam (Vquit)

ClinicalTrials.gov NCT#: NCT05162911

Funding Source: National Cancer Institute (R01CA240481)

Principal Investigators: Donna Shelley MD, MPH & Nam Nguyen, MD, DrPH

Lead Statistician: Charles M. Cleland, PhD

CLINICALTRIALS.GOV NUMBER: NCT05162911

DATE: AUGUST 16, 2023

Statistical Analysis Plan with Sample Size Calculation

1. Sample size

1.1. Sample size determination and assumptions

1.1.1. Primary outcome. The primary outcome is 7-day point prevalence biochemically confirmed (carbon monoxide (CO)<8 ppm) tobacco abstinence assessed at 6-month follow up.

1.1.2. Assumed outcome value in intervention groups. The study compares three conditions: Arm 1, referral to the Vietnam National Quitline (Quitline), Arm 2, C+SMS, and Arm 3, C+SMS+NRT. Based on prior meta-analyses and our research in Vietnam,[1-4] we estimated a modest prevalence of CO-confirmed abstinence in the Quitline group of 10%, with increased abstinence in the C+SMS (20%) and C+SMS+NRT (32%) groups.

1.1.3. Target difference in outcomes between intervention groups. As outlined above, based on prior research we estimated that C+SMS would increase abstinence rates by 10% compared with the Quitline group (OR 2.25), and that adding NRT (C+SMS+NRT) to C+SMS would increase abstinence rates by 12% (OR1.88). This pairwise contrast is the smallest expected among the three arms and is consistent with the meta-analytic evidence.

1.1.4 Statistical significance level. Family-wise error will be controlled at α = 0.05 (two-sided) for the three pairwise arm comparisons using the Holm-Bonferroni method.

1.1.5 Statistical power. We used Monte Carlo simulation (ten thousand replications) to determine the sample size needed to detect pairwise differences in the primary outcome between study arms. The power analysis is based on finding a difference between Arm 2 and Arm 3, which is expected to be the smallest difference (20% vs. 32% abstinence; OR=1.88).[1-4] Simulating data for 14 OPC sites, a total sample size of n=672 patients had at least 80% power to detect the difference between ARM 2 and 3 in conditional logistic regression analysis with Holm-Bonferroni p-value adjustments for multiple pairwise comparisons. With that sample size, power is greater than 84% to detect differences between ARM 1 and the two other arms.

1.1.6 Adjustments. We plan an intent-to-treat analysis, which will treat all participants lost to follow-up or lacking biochemical verification as assumed to be smoking. Therefore, we will not increase this sample size for dropout. Clustering will be addressed via conditional logistic regression stratified by OPC site.

1.1.7 Target sample size per trial group. The target sample size needed to detect pairwise differences in the primary outcome between study arms. The target for each arm is n=16 participants in each of 14 OPC sites, for total sample sizes of n=224 participants per arm and n=672 participants overall.

1.1.8 Software. All analyses were conducted in the R environment for statistical computing (R version 4.4.2 or later)

2. Statistical Analysis

2.1 Analysis of primary, secondary and harms outcomes

2.1.1 Main analysis methods for statistical comparisons. In an intent-to-treat analysis of all randomised participants, conditional logistic regression analysis will compare study arms on the primary outcome, 7-day point prevalence CO confirmed (<8 ppm) tobacco abstinence captured at 6-month follow up. The outcome will be regressed on indicator variables for study arm and OPC site will be the stratification variable. Pairwise comparisons of arms will be undertaken to determine which arms are significantly different. Pairwise comparisons will be adjusted for multiple comparisons using the Holm-Bonferroni method. Effect magnitudes will be based on odds ratios for the pairwise comparisons, which will be calculated by exponentiating the coefficients for pairwise contrasts.

2.1.2. Deviation from the statistical analysis plan. There are no planned deviations.

2.1.3 Prespecified vs post-hoc analyses. All analyses described in 2.1.1 are prespecified. Exploratory mediator analyses (e.g., effects of self-efficacy on abstinence rates) and Complier Average Causal Effect (CACE) per-protocol analyses to measure intervention fidelity will be reported as post-hoc/secondary analyses.

2.1.4 Effect measures and confidence intervals. Pairwise arm contrasts will be expressed as odds ratios (ORs) obtained by exponentiating model coefficients; 95 % confidence intervals will accompany all effect estimates.

2.1.5 Statistical significance level. A family-wise two-sided $\alpha = 0.05$ will be adopted for primary and secondary outcome testing.

***2.1.6 Adjusted analysis*.** Tobacco use (cigarette-only vs dual use) and level of nicotine dependence at baseline will be included as covariates. Continuous covariates will be entered untransformed unless non-linearity is detected; if so, fractional polynomials will be applied. Adjusted conditional logistic regression (and its multilevel analogue) will be used; covariates will be retained regardless of statistical significance to preserve pre-specification.

2.1.7. Multiplicity control. Pairwise arm comparisons will be adjusted for multiple testing with the Holm-Bonferroni method to control the family-wise error rate at 0.05.

2.1.8. Software. Primary and secondary analyses will be performed in R (version 4.4.2 or higher). Indirect-effect models will be fitted in Mplus with bootstrap standard errors.

2.2. Population included in analysis

The primary analysis will use an intention-to-treat set that includes every randomised participant analysed in the arm to which they were assigned. This analysis will treat all participants lost to follow-up or lacking biochemical verification of self reported smoking abstinence at 6 months as assumed to be smoking.

2.3 Handling missing data

We will conduct an ITT analysis of the main outcome which will include all randomised participants. Participants with missing outcome data (no six-month follow-up survey or no carbon monoxide confirmation of self-reported smoking abstinence at 6 months), will be classified as “not abstinent”.

2.4. Additional, subgroup and sensitivity analyses.

Differences between arms were estimated in subgroups of participants smoking at baseline: 1) cigarettes only or 2) cigarettes and waterpipe. We used the conditional logistic regression analysis described for the primary outcome analysis, adding an interaction between type of tobacco use at baseline and study arm. Estimation of this model was followed by simple pairwise comparisons between arms for each type of

tobacco use group. These pairwise comparisons in subgroups formed by the type of tobacco use variable were not adjusted for multiple comparisons.

References:

1. Jiang N, Siman N, Cleland CM, et al. Effectiveness of Village Health Worker-Delivered Smoking Cessation Counseling in Vietnam. *Nicotine Tob Res*. 2019;21(11):1524-1530. doi:10.1093/ntr/nty216
2. Moadel AB, Bernstein SL, Mermelstein RJ, Arnsten JH, Dolce EH, Shuter J. A randomized controlled trial of a tailored group smoking cessation intervention for HIV-infected smokers. J Acquir Immune Defic Syndr. 2012 Oct 1;61(2):208-15.
3. Lancaster T, Stead LF. Individual behavioural counselling for smoking cessation. Cochrane Database Syst Rev. 2017;31;3:CD001292. doi: 10.1002/14651858.CD001292
4. Stead LF, Lancaster T. Combined pharmacotherapy and behavioral interventions for smoking cessation. Cochrane Database Syst Rev. 2016 Mar 24;3:CD008286. doi: 10.1002/14651858.CD008286.pub3.

STUDY PROTOCOL

**Principal Investigator:**	Donna Shelley MD MPH<br>Population Health<br>180 Madison Ave<br>Email: donna.shelley@nyulangone.org<br>Tel: 9174944210
**Additional Investigators:**	Nam Nguyen, MD DrPH<br>Institute of Social and Medical Studies<br>810 CT1A ĐN1, Ham Nghi Street, My Dinh 2 Ward, South Tu Liem District, Ha Noi, Viet Nam<br>Email: ntnam@isms.org.vn<br>Tel: +84-4-35558288
**NYULMC Study Number:**	Study Number s19-01783
**Funding Sponsor:**	National Cancer Institute<br>Phone: 240-276-6919
**IND/IDE Number:**	NA
**Regulatory Sponsor:**	NA
**Study Product:**	Implementing Tobacco Use Treatment in HIV Clinics in Vietnam
**Study Product Provider:**	NA
**ClinicalTrials.gov Number**	NCT05162911

**Version number**	**Version Date**	**Summary of revisions made**
**1.0**	**11 FEB 2020**	**Original**
**2.0**	**2 MAR 2020**	• We have revised the protocol as per the comments<br>• Added funding source<br>• Added NRT information sheet in protocol section<br>• Revised medical clearance form in protocol section<br>• Added fidelity checklist in protocol section<br>• Revised written consent forms<br>• Revised patient waiver of consent for the focus groups.<br>• Added DSMB charter<br>• Added IRB memo from IRB in Vietnam
**3.0**	**30 MAR 2020**	• We have edited the protocol to note that materials that are not ready to be provided will be used only after NYULH and the local site IRB approval are obtained for these documents.<br>• We have created and added the key information sheet<br>• Focus group consent- We have added the risk which is breach of confidentiality
**4.0**	**15 Sept 2020**	• We have edited the protocol to add individual patient interviews to aim 1<br>• Waiver of written consent for interviews was added to the focus group waiver form
**5.0**	**16 Dec 2021**	• We have edited the patient interview guide and consent<br>• We added the provider interview guide and consent We edited the NRT type from patch to gum (Gum will be used instead of a patch because gum is commercially available to patients post intervention while the patch is not.)

		• We edited the NRT information sheet to reflect change from patch to gum<br>• We have edited the number of provider interviews from 8 to 12.
**6.0**	25 Feb 2021	• Removed details related to focus groups proposed under aim 1. It was decided that individual interviews were more appropriate to obtain the necessary data. Focus groups were therefore removed from the protocol.<br>• Removing focus groups required increasing individual interviews with patients in order to reach saturation. The number of individual interviews were estimated to be 15-20; these have been increased to 24. Participant totals changed to reflect this change.<br>• Data collection was modified to reflect that the AIM 2 patient baseline survey will be administered either in person or by telephone; and the patient eligibility screener may be collected on paper or REDCap. We are remaining flexible to accommodate different characteristics of the study sites and staffing.<br>• We changed the location of the study to Hanoi only. This change is due to COVID restrictions which limit travel within the country and reduction in budget.<br>• The definition of standard care for the intervention was changed from 3As (Ask, Advise, Assist)+ referral to 2As (Ask, Advise) + referral. The text and figures were edited to reflect this change. This change was made in response to results of the formative data that demonstrated that physicians did not have time to provide more intensive assistance (the 3rd A)<br>• Recruitment and enrollment information was edited to account for changes based on the formative research findings.<br>• The Quitline referral process was changed in order to increase the likelihood that patients will receive counseling. Patients will now be called by the Quitline rather than having to initiate the call.<br>• Data protection around Quitline referral process was added.<br>• The medical clearance form that listed the conditions that require precautions when prescribing NRT was removed; questions were moved to the screening for eligibility form, which is completed by a nurse at each clinic. We have deleted uncontrolled hypertension and unstable angina based the current recommendations on risks of NRT.<br>• The number of weeks that NRT will be provided was changed from 4 to 6 weeks. This was because the cost of gum is less than the patch which allows us to now provide the NRT for the recommended minimum number of weeks of treatment.<br>• We are adding a pilot study in Aim 1<br>• We are adding a text message protocol to enhance the counseling intervention. Patients randomized to arm 2 and 3 of the RCT will receive 2 messages per day for

		8 weeks and then one per day until end of treatment which is 12 months.
**7.0**	October 19	• A set of cost questions was added to the patients baseline survey<br>• Added a fourth aim that will study the relationship between food insecurity and tobacco use among people living with HIV/AIDS in Vietnam. This aim was added as part of a supplement that the team received.<br>• Increased the sample size by 75 from 672 to 747 to include 75 non-smokers as part of Aim 4.<br>• Added details about Aim 4 to the recruitment, enrollment, consent, and statistical considerations sections.<br>• Revised OPC eligibility: patient minimum per site was changed from 250 to 240. A minimum number of providers per site was added, 4 providers per site to ensure that we had enough sites to enroll.<br>• Revised the protocol for the three arms to go back to the original 3As (Ask, Advise, Assist) vs 2As. This was based on findings from the formative assessment. Revised the workflow to reflect the RA obtains consent from the patient rather than the clinic nurse and that the RAs will be onsite to complete all consent and baseline survey forms.
**8.0**	December 29 2022	• Added bullet point added to patient eligibility inclusion criteria to address any potential double enrollment from any patient who participated in the intervention during wave 1 so they are not enrolled during wave 2.<br>• Removed 12-month patient follow up survey, the 6-month patient follow up survey provides sufficient information on intervention completion.
**9.0**	August 16 2023	• Updated all mentions of conducting sustainability assessment at 12 months to conducting it at 18 months because we needed additional time after study completion to adequately assess sustainability.

Table of Contents

Statement of Compliance 6
List of Abbreviations........ 6
Protocol Summary 7
Schematic of Study Design........ 8
1. Key Roles........ 9
2. Background and Scientific Rationale........ 9
2.1 BACKGROUND........9
2.2 DESCRIPTION OF MEDICATION 9
2.2.1 Dose and Rationale........9
2.3 RATIONALE 10
2.4 POTENTIAL RISKS AND ENDPOINTS 10
2.4.1 Potential Risks 10
2.4.2 Potential Benefits 10
3. Objectives 10
4. Study Design and Endpoints 10
4.1 DESCRIPTION OF STUDY DESIGN 10
4.2 STUDY ENDPOINTS 11
4.2.1 Primary Study Endpoints........11
4.2.2 Secondary Study Endpoints........11
5. Study Enrollment and Withdrawal 11
5.1 INCLUSION CRITERIA 11
5.1.1 STUDY SITE ELIGIBILITY 11
5.1.2 PATIENT INCLUSION CRITERIA 11
5.1.3 HEALTH CARE PROVERS AND STAFF INCLUSION CRITERIA 11
5.2 EXCLUSION CRITERIA 11
5.2.1 PATIENT EXCLUSION CRITERIA 11
5.2.2. Employee Exclusion Criteria........11
5.3 VULNERABLE SUBJECTS........12
5.4 STRATEGIES FOR RECRUITMENT 12
5.4.1 AIM 1 AND 3 12
5.4.2 AIM 2 AND 4 12
5.5 DURATION OF STUDY PARTICIPATION........12
5.6 TOTAL NUMBER OF PARTICIPANTS AND SITES 12
5.7 PARTICIPANT WITHDRAWAL OR TERMINATION 12
5.7.1 Reasons for Withdrawal 12
5.7.2 Handling of Withdrawal 13
5.8 PREMATURE TERMINATION OR SUSPENSION OF STUDY 13
6. Study Drug 13
6.1 STUDY DRUG DESCRIPTION 13
6.1.1 Acquisition........13
6.1.2 Packaging, and Labeling........13
6.1.3 Storage 13
6.1.4 Dosing, Route of Administration, and Duration of Therapy 13
6.1.5 Dose Adjustments 13
6.1.6 Tracking Dose 13
6.2 STUDY AGENT ACCOUNTABILITY PROCEDURES 13
6.3 STUDY INTERVENTION........14
6.3.1 Randomization 14
6.3.2. Administration of Intervention 14
6.3.3 Procedures for Training Interventionalists and Monitoring Intervention Fidelity........15
6.3.4 Assessment of Subject Compliance with Study Intervention........16
7. Study Procedures and Schedule........ 16
7.1 STUDY SCHEDULE 16

7.1.1 Screening ... *16*
7.1.2 Enrollment/Baseline ... *17*
7.1.3 Study Visits ... *17*
7.1.4 Methods and Procedures ... *17*
7.1.5 Withdrawal/Early Termination Visit ... *17*
7.1.6 Unscheduled Visit ... *18*
7.1.7 Participant Access to Study Agent at Study Closure ... 18
7.1.8 Prohibited Medication ... 18
7.1.9 Final Study Visit ... *18*
8. Assessment of Safety ... 18
8.1 Specification of Safety Parameters ... 18
8.1.1 Definition of Adverse Events (AE) ... *18*
8.1.2 Definition of Serious Adverse Events (SAE) ... *18*
8.1.3 Definition of Unanticipated Problems (UP) ... *19*
8.2 Classification of an Adverse Event ... 19
8.2.1 Severity of Event ... *19*
8.2.2 Relationship to Study Agent ... *19*
8.2.3 Expectedness ... *19*
8.3 Time Period and Frequency for Event Assessment ... 19
8.4 Reporting Procedures – Notifying the IRB and Sponsor ... 20
8.4.1 Adverse Event Reporting ... *20*
8.4.2 Serious Adverse Event Reporting ... *20*
8.4.3 Unanticipated Problem Reporting ... *20*
8.4.4 Reporting of Pregnancy ... *21*
8.5 Reporting Procedures – Notifying the Study Sponsor ... 21
8.6 Reporting Procedures – Notifying the FDA ... 21
8.7 Halting Rules ... 21
8.8 Safety Oversight ... 21
9. Statistical Considerations ... 21
9.1 Statistical and Analytical Plans and Methods (SAP) ... 21
9.2 Statistical Hypotheses ... 23
9.3 Planned Interim Analysis ... 23
10. Source Documents and Access to Source Data/Documents ... 23
11. Quality Assurance and Quality Control ... 23
12. Ethics/Protection of Human Subjects ... 24
12.1 Informed Consent Process ... 24
12.2 Participant and Data Confidentiality ... 25
13. Data Handling and Record Keeping ... 25
13.1 Data Collection and Management Responsibilities ... 25
13.2 Study Records Retention ... 25
13.3 Protocol Deviations ... 25
13.4 Publication and Data Sharing Policy ... 26
14. Study Finances ... 26
14.1 Funding Source ... 26
14.2 Costs to the Participant ... 26
14.3 Participant Reimbursements or Payments ... 26
15. Study Administration ... 26
15.1 Study Leadership ... 26
16. Conflict of Interest Policy ... 26
17. References ... 27

STATEMENT OF COMPLIANCE. This study will be conducted in accordance with the Code of Federal Regulations on the Protection of Human Subjects (45 CFR Part 46), 21 CFR Parts 50, 56, 312, and 812 as applicable, any other applicable US government research regulations, and institutional research policies and procedures. The International Conference on Harmonization ("ICH") Guideline for Good Clinical Practice ("GCP") (sometimes referred to as "ICH-GCP" or "E6") will be applied only to the extent that it is compatible with FDA and DHHS regulations. The Principal Investigator will assure that no deviation from, or changes to the protocol will take place without prior agreement from the sponsor and documented approval from the Institutional Review Board (IRB), except where necessary to eliminate an immediate hazard(s) to the trial participants. All personnel involved in the conduct of this study have completed Human Subjects Protection Training.

ABBREVIATIONS

**AE**	Adverse events
**AIDS**	Acquired Immunodeficiency syndrome
**ART**	Antiretroviral therapy
**CACE**	**Complier Average Casual Effect**
**CIOMS**	Council for International Organizations of Medical Science
**CO**	Carbon monoxide
**CRF**	Case report form
**FDA**	Food and Drug Administration
**DSMB**	Data safety monitoring board
**GCP**	Guideline for Good Clinical Practice
**HIPAA**	Health Insurance Portability and Accountability Act of 1996
**HIV**	Human Immunodeficiency Virus
**ISMS**	Institute of Social and Medical Studies
**KI**	Key informant interviews
**MI**	Myocardial infarction
**NIDA**	National Institute on Drug Abuse
**NIH**	National Institutes of Health
**NRT**	Nicotine replacement therapy
**NYU**	New York University
**OPC**	Outpatient clinic
**OTC**	Over the counter
**PHI**	Protected health information
**PI**	Principal investigator
**PLWH**	People living with HIV
**QC**	Quality control
**RA**	Research assistant
**RCT**	Randomized controlled trial
**SAC**	Scientific Advisory Committee
**SAE**	Serious adverse event
**SAP**	Statistical and analytic plans
**SC**	Standard care
**SoM**	School of Medicine
**TUT**	**Tobacco use Treatment**
**UP**	Unreported problem
**VHW**	Village health worker
**WHO**	World Health Organization

PROTOCOL SUMMARY

Title	Implementation of tobacco use treatment in HIV clinics in Vietnam
Brief Summary	The primary objective of this study is to conduct a 3-arm randomized controlled trial (RCT) that compares the effectiveness of three multi-component interventions that are embedded in HIV outpatient clinics (OPCs): 1)3As+Referral (Standard Care (SC)): Ask about tobacco use, Advise to quit, Assist with brief counseling and Refer to Viet Nam's national Quitline; 2) AAA+ +Counsel (Counsel=6-session cessation counseling intervention adapted for patients living with HIV/AIDS (PLWH) and delivered by a trained, onsite nurse; and 3) AAA+Counsel+N (N=nicotine replacement therapy (NRT)). The main outcome is biochemically validated 6-months smoking abstinence. We will recruit and randomize 672 patients across 13 outpatient clinics (OPCs) (48 per site, 16 per arm). We will also recruit 75-nonsmokers to participate in a single survey to assess food safety. Therefore, the total sample = 747 patients.
Phase	Phase 3
Objectives	The primary objective of this study is to conduct a 3-arm RCT that compares the effectiveness of three multi-component interventions to increase smoking abstinence among PLWH.<br><br>The secondary objective is to assess factors (health care setting, provider, and patient level factors) that may influence tobacco cessation, and sustainability of the interventions tested.<br><br>The primary objective of the supplement is to examine the difference in household food insecurity among people living with HIV and the relationship between food insecurity and tobacco use in Vietnam.
Methodology	The is a 3-arm unblinded RCT
Endpoint	The primary endpoint is carbon monoxide confirmed smoking abstinence at 6 months.
Study Duration	Five years
Participant Duration	Patients and providers will be enrolled for 2 years
Duration of IP administration	Nicotine gum (for those randomized to Arm 3 in the RCT) will be given for 6 weeks.
Population	We are recruiting tobacco users who are patients at one of the 13 participating OPCs. All patients are Vietnamese. We will recruit women and men. For the research supplement (AIM 4) we will recruit non-smokers from the 13 OPCs.
Study Sites	13 HIV outpatient clinics in Hanoi
Number of participants	941 (includes providers and patient participants)
Description of Study Agent/Procedure	The intervention being tested in the RCT is behavioral only in ARM 1 and 2 and in ARM 3 patients will also receive nicotine gum for daily use for 6 weeks.
Reference Therapy	NA. There is no placebo. This is an effectiveness study using an over the counter drug.
Key Procedures	NA

Statistical Analysis	The primary analytic approach used to compare study arms is conditional logistic regression. This fixed effects approach was chosen as primary because of the modest number of sites and the fact that they are not very heterogeneous. Because some methodologists have advocated mixed effects models even when the number of sites is small, we will use a multilevel logistic regression model as an alternative, sensitivity analysis approach. In an intent-to-treat analysis of all study entrants, conditional logistic regression analysis will compare study arms on the primary outcome, 7-day point prevalence CO confirmed (<8 ppm) tobacco abstinence captured at 6-month follow up. The outcome will be regressed on indicator variables for study arm and OPC site will be the stratification variable. Pairwise comparisons of arms will be undertaken to determine which arms are significantly different. Pairwise comparisons will be adjusted for multiple comparisons using the Tukey method. Effect magnitudes will be based on odds ratios for the pairwise comparisons, which will be calculated by exponentiating the coefficients for pairwise contrasts. Secondary cessation outcomes, HIV-related outcomes, hypothesized mediators (e.g., risk perceptions, self-efficacy), and the food insecurity outcomes also will be analyzed with conditional logistic regression, or a different fixed effects regression appropriate to the type of outcome variable.

SCHEMATIC OF DESIGN. This research has three phases. For Aim 1 we will conduct a formative

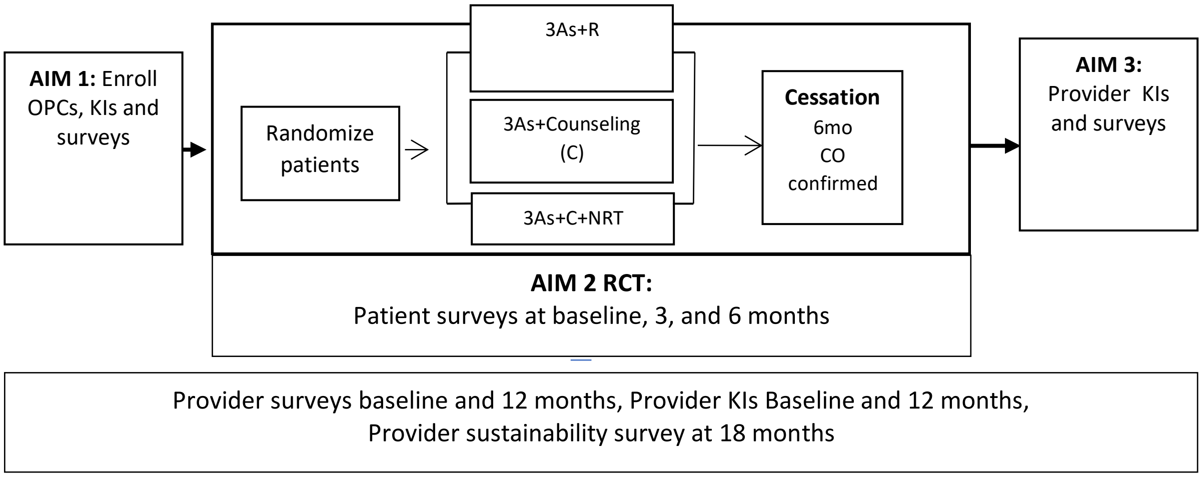

assessment to inform further modifications to the intervention components. Participants in Aim 1 will be patients of the outpatient clinics (OPC) or health care providers who work there. Patients who use tobacco will be asked to participate in individual interviews (n=24). We will conduct key informant interviews with health care providers from 3 OPCs (n=28) to adapt the intervention to the OPC clinical context. We will then conduct a pilot test of ARM 1 and 3 in one OPC with 16 patients. The recruitment and enrollment process will be the same as described for Aim 2. For Aim 2 we will conduct a three-arm randomized controlled trial (RCT) to compare the effectiveness of three interventions aimed at increasing tobacco cessation rates among people living with HIV/AIDS (PLWH). Patients (n=672) will be recruited and enrolled from the 13 OPCs. All enrolled patients will complete a baseline survey in person. This survey will be administered once eligibility is established and consent is obtained. The consent and survey will last 30 minutes. Follow up surveys will occur at 3- and 6-months after enrollment. Follow up surveys will be conducted by telephone. At 6-months follow up, patients who report smoking abstinence will be asked to come in person to the OPC and will complete a carbon monoxide test to validate self-report. We will conduct baseline and 12-month surveys with all health care providers in the study sites in person (n=98). For Aim 3 we will conduct 12-month post intervention interviews and an 18-month survey to assess factors associated with implementation effectiveness and potential for sustainability. For Aim 4 we will enroll 75 more patients who are non-smokers for a total of (n=747) patients and assess the relationship between food insecurity and tobacco use among PLWH (75 smokers and 75 non-smokers), which includes administering the Household Food Insecurity Access Scale (HFIAS) survey to patients at baseline and 6-months.

1. KEY ROLES

Principal Investigator
Donna Shelley, MD MPH
Professor, Public Health Policy and Management
NYU School of Global Public Health
Professor, Population Health
Department of Population Health
NYU School of Medicine
715 Broadway
NY, NY 10012
Donna.shelley@nyu.edu

Principal Investigators
Nam Nguyen, MD, DrPH
Director-Institute of Social and Medical Studies
19, Alley 6, Dong Bat Street, My Dinh 2 Ward, South Tu Liem District,
Ha Noi, Viet Nam Phone/fax: 84-4-35558288
ntnam@isms.or.vn
www.isms.org.vn

Co-Investigator
Charles Cleland, PhD
Division of Biostatistics
Department of Population Health
NYU School of Medicine
180 Madison Ave
NY, NY 10016
Charles.cleland@nyulangone.org

2. BACKGROUND AND SCIENTIFIC RATIONALE.

2.1 BACKGROUND. Despite the existence of effective tobacco dependence treatments, cigarette smoking remains the leading cause of preventable morbidity and mortality in Viet Nam.[1] Viet Nam also has one of the highest HIV burdens in the Asia and Pacific region. Over 300,000 adults aged 15-49 are HIV infected, with 10,195 new HIV infections and 6,130 new cases of AIDS in 2015. In response to the epidemic, Viet Nam has rapidly expanded treatment services through a robust network of outpatient clinics (OPCs) for People Living with HIV/AIDS (PLWH) that provide free access to antiretroviral therapy (ART), testing and counseling. However, there are no tobacco cessation programs in OPCs.

2.2 DESCRIPTION OF MEDICATION

***2.2.1* Dose and rationale**. We will be using nicotine gum which is an FDA approved medication for smoking cessation. This medication is over the counter and therefore poses minimal risk. We will be providing gum of 2 mg for 6 weeks which is consistent with trials of efficacy.[2,3] Despite the burden of disease related to tobacco use, there is a lack of evidence for the long-term effectiveness of tobacco cessation interventions for PLWH. Inadequate implementation of evidence-based tobacco use treatment interventions in HIV treatment settings exacerbates the health and economic burden caused by the high prevalence of smoking in Viet Nam. Developing and implementing effective tobacco cessation interventions is critical for optimizing treatment outcomes and survival for PLWH who use tobacco.

2.3 POTENTIAL RISKS AND BENEFITS

***2.3.1* Potential risks**. This study poses minimal risk to both patients and employees enrolled in the study. Loss of confidentiality is the greatest potential risk to study subjects. All participants will receive an identification number that will be used for all forms, transcripts, surveys and intervention materials. For patients, we are using two behavioral interventions and nicotine gum. The nurse conducting enrollment will assess risk factors related to the use of nicotine replacement therapy (NRT) that would exclude patients from enrolling. Patients with a recent history of myocardial infarction (MI) (two weeks), serious underlying arrhythmias, and patients who are pregnant or nursing will not be offered NRT. It is important to note that the nicotine gum is an over-the-counter medication, with proven safety, and with minimal potential side effects. In addition, there are no significant interactions between NRT and HIV treatments (http://www.hivdruginteractions. org/PrintableCharts.aspx). Recent changes in FDA labeling also confirm that it is safe for tobacco users to use NRT while continuing to smoke. The FDA label for over the counter (OTC) NRT now says that: "concomitant use of OTC NRT products with cigarettes or with other nicotine-containing products does not raise significant safety concerns". Employees in the participating health clinics will be asked to sign consent which will explain the voluntary nature of their participation and that they can withdraw their participation at any time without consequences in terms of risk to employment. Employee data will be treated as described above to ensure that their responses to survey questions and interviews remains confidential and cannot be linked to any employment records. The enrollment and consent procedures to ensure that there is no coercion are described below in section 12.1.

***2.3.2* Potential benefits.** Patients who receive the counseling and/or nicotine gum may quit using tobacco.

3. OBJECTIVES. The objectives of the main study are 1) to conduct a formative assessment to guide adaptations of the current intervention components to optimize implementation of tobacco dependence treatment, 2) to conduct a 3-arm randomized controlled trial (RCT) that compares the effectiveness of three multi-component interventions that are embedded in HIV OPCs, and 3) to assess barriers and facilitators to implementation and potential for sustaining gains. (See schematic above). The objectives of the supplement are 1) to estimate the difference in household food insecurity among PLWH who smoke compared with PLWH who are non-smokers at baseline. 2) to examine mechanisms linking smoking and household food insecurity among PLWH at baseline and 6-months follow-up, and 3) to assess the impact of a reduction in tobacco use and tobacco cessation on measures of food Insecurity at 6-month follow-up.

4. STUDY DESIGN and ENDPOINTS

4.1 DESCRIPTION OF STUDY DESIGN. This is a phase 3 pragmatic comparative effectiveness study. **AIM 1:** We will collect pretrial data using individual interviews with tobacco users to inform further tailoring of the intervention for HIV+ smokers and individual interviews (n=28) with health care providers in three OPCs. We will then conduct a pilot test of ARM 1 and 3 in one OPC with 16 patients. **AIM 2:** We will then conduct a 3-arm RCT to compare the effectiveness of three multi component interventions: 1) Ask, Advise, Assist (AAA) + Refer to the national smokers' Quitline); 2) AAA+Counseling (Counsel) which adds referral to an onsite health worker for 6-sessions of cessation counseling tailored to PLWH; and 3) Addition of 6-weeks of nicotine gum (AAA+Counsel+N). Only ARM 3 participants will receive NRT. ARM 2 and 3 will receive two text messages daily for 8 weeks and then 1 per day for 4 weeks to reinforce the counseling protocol. A total of 747 participants will be recruited in 13 outpatient HIV clinics (OPCs) in Hanoi and randomized into one of the three arms (48 smokers per site, 16 per arm; about 5 non-smokers per sitecWe will conduct baseline in person surveys with all providers (n=98).

AIM 3: We will conduct 12-month post intervention interviews and 18-month surveys with healthcare providers and other key informants to assess factors associated with implementation effectiveness and potential for sustainability.

AIM 4: We will collect data on food insecurity form 75 non-smokers and 672 smokers at baseline and 6-months.

4.2 STUDY ENDPOINTS*4.2.1 Primary*. The primary endpoint is carbon monoxide confirmed smoking abstinence at 6 months.

4.2.2 Secondary. The secondary patient outcomes are self reported smoking cessation and reduction in number of cigarettes per day, and hypothesized mediators of the main outcome (risk perception, attitudes, norms and self-efficacy) at 3 and 6 months.

5. STUDY ENROLLMENT AND WITHDRAWAL.

5.1 INCLUSION CRITERIA.

5.1.2. Study site eligiblity. We will recruit 13 OPCs from one city, Hanoi. The sites are very homogeneous in terms of staffing and infrastructure. Therefore, site eligibility will be based solely on the number of active patients to ensure adequate recruitment (>240 patients) and at least 4 providers per site. The CDC of Hanoi will assist with site recruitment.

5.1.3. Patient inclusion crieria. For Aim 1-3 men and women who are $\geq$18 , patients in the HIV OPCs, current tobacco users, live in Hanoi and reachable by phone are eligible. All patients of the OPCs are HIV+. We expect fewer women to be enrolled in all phases of this research because about 10-20% of patients in the OPCs are female, and their smoking prevalence is low (<2%). There are no enrollment restrictions based upon race and ethnicity. For the supplement we will enroll men and women who are >18 and patients in the HIV OPCs who are not current tobacco users.

5.1.4. Health care providers and staff inclusion criteria. All providers and staff currently working at the OPC will be eligible.

5.2 EXCLUSION CRITERIA

5.2.1. Patient Exclusion Criteria The few individuals for whom there is a need for precaution in using NRT will be excluded

- recent myocardial infarction (2 weeks)
- serious underlying arrhythmias
- patients who are pregnant or nursing
- any patient who participated in the study during wave 1 at OPC site 1-5 and then transferred to OPC site 6-13 during wave 2

We will also exclude those who are unable to demonstrate capacity for consent and patients already enrolled in a tobacco use treatment program.

Our pilot data indicate that about half of tobacco users in these sites are dual users (cigarettes and waterpipe); only about 12% use waterpipe alone. VQUIT found that dual users who received VHW counseling had 6-month CO confirmed quit rates that were comparable to patients who used cigarettes only. Therefore, we will not exclude dual users. We will exclude waterpipe only users because the intervention is based on effectiveness data for dual and cigarette users and there is a lack of evidence for efficacy or effectiveness of NRT use in this population.

5.2.2. Employee exclusion criteria. Employees who are unwilling to sign consent will be excluded.

5.3 VULNERABLE SUBJECTS. Employees are considered a vulnerable population. Research staff who work for the research institute (ISMS) will be responsible for consenting employees. Research staff are trained to emphasize that participation is voluntary and employees' decision will not affect their job evaluation, promotions and work-related assignments.

5.4 STRATEGIES FOR RECRUITMENT.

5.4.1. AIM 1. We will work with the Hanoi CDC who will contact OPCs to inform potential study sites for about the study aims and obtain permission to have ISMS research staff contact them to provide additional information and assess eligibility. We will then reach out to set up a meeting with the medical directors to describe the study. If they are interested in participating the site will be enrolled. For individual interviews, patients will be screened at one of the three OPCs that agree to participate in the Aim 1 data collection. They will be screened for current tobacco use in the waiting room, asked to participate if they meet eligibility criteria, and given a date and time for the interviews. Verbal consent will be obtained prior to completing the screening questionnaire. Health care providers who work in the participating OPCs will be asked to attend a brief meeting in that site. Providers will be told about the project, including the data collection. They will be asked if they are willing to participate in the KIs at baseline (AIM 1). Those who agree will be contacted by a Research Assistants (RA) to set up a time for KI and obtain verbal consent. The pilot study will use the Aim 2 RCT recruitment approach. (see below)

***5.4.2.* AIM 2.** 13 OPCs will be enrolled in the study as per the protocol described above. We propose to enroll 672 patients (48 per site). Patients arriving for previously scheduled clinic visits will be asked about their tobacco use by a trained nurse who is responsible for checking patients in at the time of their visits. The nurse will describe the study and screen for eligibility. Eligible patients will be introduced to the RA who will be onsite. The RA will read the consent and if the patient is willing to participate and signs the consent form the RA will enroll them, complete the baseline survey and randomize to one of the three study arms. Health care providers will again be asked to participate in a brief meeting to discuss the details of the RCT. We will then use the same recruitment process as in AIM 1. However, at this time we will be obtaining written consent to complete baseline and 12-month surveys and a post intervention KI at 12 months.

5.4.3. AIM 3. At the time of enrollment in AIM 2, providers will also be asked to participate in the KIs 12 months after the intervention period. Consent will be obtained at the time they enroll in AIM 2. For the 18-month sustainability survey, written consent will be obtained at the time they are approached to participate in the survey.

5.4.4. Aim 4. We will enroll 75 patients who are non-smokers using the same strategy as AIM 2.

5.5 DURATION OF STUDY PARTICIPATION. Patient individual interview participants will be asked to participate for the single session. Providers and patients who participate in the RCT will be enrolled for 2 years. Patients who are non-smokers (n=75) participating in the diversity supplement study will be enrolled for six months.

5.6 TOTAL NUMBER OF PARTICIPANTS AND SITES. For Aim 1, we will conduct 24 patient interviews and 28 provider interviews across 3 OPCs. We will recruit 16 patients in the one OPC that will serve as the pilot study site. For Aim 2 we include 672 patients (48 patients per site, 16 per arm). Surveys will be conducted, at baseline, with all health care providers in the 13 sites in person (n=98). For Aim 3, we will repeat surveys and KIs among the 48 providers with 2 health care providers per site, 13 sites, (n=28) at 12-months post intervention, and will separately conduct a sustainability survey at 18-months with the same providers. For Aim 4, we will include 75 patients (at least 5 patients per site).

5.7 PARTICIPANT WITHDRAWALS OR TERMINATION.

***5.7.1*. Reason for withdrawal.** This is a minimal risk study so we do not expect participants to withdraw for a safety-related issue. However, participants (providers and patients) are free to

withdraw from participation in the study at any time upon request without any impact on their ability to continue to receive care or to be employed.

5.7.2. Handling of withdrawal. We will track all withdrawals and reasons for withdrawals. Participation is voluntary and all study participants may withdraw from the study at any time without penalization. However, patient participants in the Aim 2 RCT will be encouraged to continue to participate in the primary outcome assessment of smoking cessation (i.e., follow up telephone surveys and if reported quitting, return for subsequent carbon monoxide assessment), unless a participant withdraws consent. Patients and providers enrolled in the longitudinal monitoring of cessation (patient surveys) and provider-level constructs (provider surveys and KIs) are considered lost to follow up after 4 attempts to contact them by phone. No replacement of participants who withdraw or discontinue early will occur. Date and reason for withdrawal will be recorded.

5.8 PREMATURE TERMINATION OR SUSPENSION OF STUDY. This study may be temporarily suspended or prematurely terminated if the Ministry of Health retract their current strong support for this project. Written notification, documenting the reason for study suspension or termination, will be provided by the suspending or terminating party to the study principal investigators (PI) (Nguyen/Shelley). If the study is prematurely terminated or suspended, the PIs will promptly inform the IRB and will provide the reason(s) for the termination or suspension. Circumstances that may warrant termination or suspension include, but are not limited to: a) Insufficient compliance to protocol requirements, b) Loss of institutional political support, c) Determination that the study procedures are too invasive. The study may resume once concerns about safety, protocol compliance, data quality are addressed and satisfy the sponsor and/or IRB.

6. STUDY DRUG

6.1 STUDY DRUG DESCRIPTION. Nicotine gum has been FDA approved for over 20 years. They are available as over the counter medications. Nicotine gum is a form of nicotine replacement therapy that delivers a specific dose of nicotine to people who use tobacco in order to reduce withdrawal symptoms when they are trying to quit. Gum is placed in the mouth and chewed multiple times per day.

6.1.1. Acquisition: Nicotine gum will be purchased in Vietnam.

6.1.2. Packaging and labelling**.** The gum comes in boxes of 12 tablets, each tablet contains 2 mg of nicotine. One box can be used for 1 to 1.5 days.

6.1.3 Storage. NRT is stored in locked cabinets at ISMS and at the pharmacy of each OPC. The medication does not require any special temperature or storage conditions.

6.1.4 Dosing, route of administration and duration of therapy. All patients will receive 6 weeks of 2 mg nicotine gum.

6.1.5 Dose adjustments**.** All patients will be given a one-page information sheet that explains potential side effects and includes the site study PI contact information. If a patient who is experiencing side effects associated with a need to reduce the dose (i.e., palpitations, nausea, light headedness) we will change the number of pieces of gum used per day. This is extremely rare and usually due to the fact that the patient is continuing to smoke while using the gum. Patients will be advised, again, to not smoke while using NRT.

6.1.6. Tracking dose. The counselors have a tracking form to assess use of NRT. This form is completed at each counseling session and includes three questions that ask if the patient used the NRT provided, if the patient used the gum since the last session as directed, how many pieces of nicotine gum they used since their last session and how many boxes were used.

6.2 STUDY AGENT ACCOUNTABILITY PROCEDURES. ISMS research staff will be responsible for dispensing the NRT to OPC pharmacies. Subjects will receive 6 weeks of gum starting at the first week of counseling and dispensed at each follow up session up to 6 weeks, We will order enough for the number of subjects we plan to enroll in ARM 3 which includes NRT. RAs will sign out the number of boxes they are taking to the field during the enrollment period and give NRT to the

pharmacist. When providing the gum to patients, the pharmacists will input patient name, date, time, the patient's ID, # boxes provided, the lot number for each box and expiration date on the NRT tracking form. The patient will sign that form to indicate that they have received the medication to the patient. All NRT not dispensed (because of fewer enrollees on a particular day) will be returned to the ISMS office and locked in a storage cabinet. Regular study drug reconciliation will be performed to document drug assigned, drug consumed, and drug remaining. See Section 6.3.6 for how counselors will track patient NRT use. . Reconciliation will be logged on the drug reconciliation form, and signed and dated by the study team. At the end of the study period we will destroy all unused medication as per safety instructions for discarding NRT gum.

6.3. STUDY INTERVENTION ARMS

6.3.1. Randomization

Once RAs enroll a patient in the study and the baseline survey is completed (i.e., signed consent), they will use a preprogrammed system in REDCap to randomize patients to one of the three study arms. Our biostatistician will create the REDCap database that will prompt randomization to the appropriate condition.

***6.3.2. Administration of Intervention*.** Below we describe the protocol for the RCT intervention components. The materials related to the intervention described below (i.e., brochures, counseling manual and counseling session tracking booklet) will only be used after NYULH and local site IRB approval are obtained for these documents.

***ARM 1.* Ask, Advise, Assist (AAA) and Refer.** The research team will send patient contact information to the Quitline which will proactively call the patient. We will train providers and develop a tool kit that will include information for providers and patients.

***ARM 2:* AAA plus referral to onsite counselor (Counsel). Multisession counseling:** We will provide a packet for each patient randomized to ARM 2 or 3 to the designated nursing staff that has been trained to provide the counseling intervention that includes all intervention handouts. The nurse counselor will receive a manual, session tracking booklet and fidelity checklist. Nurse manuals will include additional information about NRT to be reviewed with patients in ARM 3 and a document to use to track the use of the nicotine gum during each session. Our decision to train nurses is consistent with the staffing infrastructure, recommendations from the OPC directors and Provincial health directors and Dr. Nguyen's experience working in these sites. Participants will receive six sessions of in person and telephone counseling depending on their preferences **(Table 2).** There is evidence that a greater number of sessions may be associated with higher smoking abstinence rates among PLWH, but these outcomes are short-term (3 month), with a loss of effect by 6 months. Therefore, there is no definitive research on the optimal number of sessions that will lead to long-term outcomes in PLWH. Rather, the literature suggests that it is not only the number of contacts but also the timing (i.e., need for contact over a longer period of time to maintain abstinence).[4-8]Therefore, the number of sessions and timing is based on an extensive review timing (i.e., need for contact over a longer period of time to maintain abstinence).[4-8]Therefore, the number of sessions and timing is based on an extensive review of the literature, including relevant research conducted by Dr. Go (consultant) among PLWH in Viet Nam[98] and our pilot research that explored feasibility based on patient and counselor time demands.

Table 2. Counseling protocol

Sess.	Wk	Content
1	1	Build motivation, significance of quitting for HIV+ smokers, elicit triggers, quit plan
2	2	Dealing with withdrawal, managing stress, refusal skills
3	3	Managing high risk situations (e.g., other substances), coping with thoughts and feelings
4	4	Review problem solving, stress management and building social support
5	8	Maintaining commitment and motivation, anticipating and preventing slips
6	12	Successes and challenges, long term relapse prevention

Patients will receive 4 sessions in the first 4 weeks and sessions at 8 and 12 weeks that focus on maintaining long-term abstinence **(Table 2).** The first session will last 45 minutes and the following sessions about 30 minutes each. The first session will be scheduled within one week, with sessions 2 through 4 scheduled weekly and the two additional sessions scheduled for weeks 8 and 12. The counseling manual, tailored to PLWH in Viet Nam, will integrate motivational interviewing techniques with a social cognitive skills building approach, both of which have been found effective in increasing long-term abstinence in general populations of smokers and short-term abstinence in PLWH and in smokers enrolled in the VQUIT counselingintervention.[4-15]

ARM 3: AAA+Counsel+N (Nicotine gum). At the end of the first session patients will be taken to the pharmacists who will give participants the first week supply of nicotine gum and a one-page instruction sheet on use, potential side effects and who to call if they have concerns. ARM 2 and 3 will receive two text messages daily for 8 weeks and then 1 per day for 4 weeks to reinforce the counseling protocol.

6.3.3. Procedures for Training Interventionalists and Monitoring Intervention Fidelity.

***6.3.4.* Clinician Training**: All clinical and support staff will attend a 2-day training that includes building skills to screen all patients for tobacco use, offer brief cessation advice and refer patients to the Quitline. We will conduct a one-day booster training after 3 months to reinforce treatment protocols and concepts. *In VQUIT, we achieved 98% attendance for the 3-day training.* The curriculum for this proposed study is being adapted from the VQUIT training which was informed by the US Guideline: *Treating Tobacco Use and Dependence,*[10] *additional literature on effective interventions for TUT*[8-10] and the World Health Organization's (WHO) guideline: "Strengthening Health Systems for Treating Tobacco Use".[16] Based on findings from AIM 1, we will further adapt the training to address HIV-specific consequences of tobacco use and barriers to quitting. The training will be conducted by Drs. Shelley and Nguyen. We will evaluate the adequacy of training with *pre-and post-surveys at the initial and booster trainings.* ISMS staff and Dr. Nguyen will make the training available online to address potential staff turnover (which is rare in these sites) and to increase potential for dissemination in HIV OPCs.

***6.3.5.* Counselor Training.** Two nurses from each site will participate in a 4-day training with the that will include an interactive approach using videotaped examples of counseling techniques and role-playing to ensure proficiency in motivational and skill building techniques. The counselor training is based on the curricula that was adapted for VQUIT and will be further tailored for this study (AIM 1). Counselors will receive monthly supervision by trained ISMS staff and attend a booster training one month after the initial training. Standardized training, supervision and the inclusion of a booster session will help ensure intervention fidelity.

***6.3.6.* Fidelity.** We will assess fidelity in several ways. Patient baseline and 3- and 6-months surveys will assess receipt of standard care (i.e., brief provider advise if they spoke with the Quitline). We will assess NRT use at each counseling session up to week 6). Counselors will ask patients: How many boxes of NRT did you use since the last session, if the patient used the gum since the last session as directed, and how many pieces of NRT are you chewing each day?

Fidelity to counseling manual: After each contact, counselors will also complete a fidelity checklist which is used as our primary measure. ISMS staff will review observations and checklists in supervision meetings. Additionally counseling sessions will be assessed by asking the counselors to record a random sample of sessions. A trained RA will listen to tapes and use a checklist to assess adherence to the manual.

Fidelity to treatment condition assignment: To reduce potential for contamination our REDCap database will prompt randomization to the appropriate condition and pharmacists will provide the NRT to participants to ensure only those in ARM 3 receive the medication. Given the additional training and time required to deliver counseling, we do not expect that participants assigned to ARM 1 will receive the intensive counseling we are testing in ARM 2 and 3. This was confirmed in

the pilot study. However, 3- and 6-months follow-up patient surveys will assess any use of NRT and other cessation services across all study arms.

6.3.7 Assessment of Subject Compliance with Study Intervention. As noted above, counselors will track completion of all sessions and we will track use of NRT will be tracked by both the counselors.

7. STUDY PROCEDURES AND SCHEDULE

7.1 STUDY SCHEDULE

7.1.1. Screening

AIM 1: *Individual interviews with patients*: A nurse will assess eligibility among patients that they are checking in for regularly scheduled appointment and explain that we are conducting a study and describe the purpose of the study. Patients will then be asked if they are willing to complete screening questions to assess eligibility. If eligible, the subject will be asked to choose from several dates to participate in an interview. No screening data will be kept for the purpose of the study unless subjects consent to participation. All study related activities and study data collection will occur only AFTER verbal consent is obtained. We are requiring that the interviews are audiotaped and therefore will let potential participants know that at the time they are screened to offer them an opportunity to decline at that time. However, we will again explain that the interview will be audio taped prior to beginning the interview during the verbal consent process at which time they will have another chance to opt out.

Key informant interviews with providers will be conducted in aim 1 to inform the design of the intervention. Health care workers will be told about the study at a group meeting. It will be made clear to them that participation is voluntarily, and they don't have to participate. A research coordinator will follow up with a phone call to schedule interviews. We will obtain verbal consent. Once the intervention components are finalized, we will conduct a pilot study in one OPC with 16 patients randomized to ARM 1 and 3 of the proposed RCT. Screening and recruitment will follow the approach described in Aim 2 below.

AIM 2: *Patient recruitment*: Nurses will assess smoking status at the time patients register for their visit. If patients are current smokers, nurses will briefly describe the RCT study and explain that those patients who are interested in participating will be screened for eligibility. Patients who refuse to be screened or who do not meet eligibility criteria will be provided a cessation brochure. Those patients who meet eligibility criteria will be introduced to the onsite RA. The RA will read the consent and the patient will sign a paper version in the clinic. Once they consent, the RA will conduct the baseline survey and randomize patients before their provider visit. The consent process and survey will take about 30-40 minutes.

Healthcare provider recruitment: Health care workers will be told about the study at a group meeting. It will be made clear to them that participation is voluntarily and they don't have to participate. This will be repeated at the time of the consent process. All health workers are eligible. Research staff will follow-up to contact providers and staff to make appointments for to administer the baseline and follow up surveys. Written consent will be obtained at the time of the baseline survey. Health care providers who participate in surveys will be asked to complete the survey again at 12 months post intervention. We are requiring that the interviews are audiotaped, as described above. We will let potential participants know about the audio taping at the time we describe the study to offer them and opportunity to decline at that time if that is a reason they do not want to participate. However, we will again explain that the interviews will be audio taped prior to beginning at which time they will have another chance to opt out.

AIM 3*: Provider interviews.* Health care workers who were enrolled in AIM 2 will have been consented to also participate in interviews and surveys post intervention to assess the implementation process.

AIM 4: *Patient recruitment:* non-smokers will be recruited using the methods described for recruiting smokers as detailed in AIM 2 patient recruitment above.

***7.1.2 Enrollment/Baseline*.**

AIM 1: ***Patient Interviews****:* We will confirm eligibility and then ask participants to choose an interview date. Participants will be offered an explanation of the purpose of the study, given a one-page description with a number to call if they have additional questions. We will obtain verbal consent at the time of the or interview immediately before it begins.

AIM 1 and 3: ***Provider surveys and KIs*:** We will confirm eligibility which only includes that the provider is currently working at the OPC. Providers will be asked to provide verbal consent for preintervention interviews and written consent to enroll in the longitudinal assessment (surveys and KIs). The initial survey and KI will occur at baseline and then they both will be repeated at 12 months. A separate survey to assess sustainability will be conducted at 18 months, for which written consent will be obtained before administering the survey.

AIM 2 RCT and AIM 4: Nurses will assess smoking status at the time patients register for their visit. Nurses will describe the study and explain that those patients who are interested in participating will be screened for eligibility. Patients who refuse to be screened or who do not meet eligibility criteria will be provided a cessation brochure. Those patients who meet eligibility criteria will be introduced to the study RA. The RA will obtain written consent. Once they consent, the RA will conduct the baseline survey and randomize patients. The consent process and survey will take about 30-45 minutes.

7.1.3 Study visits

AIM 1 Patients: The first and final visit for patients in AIM 1 is the interview. Patients enrolled in the AIM 1 pilot study will complete the same study visits as described below in Aim 2.

AIM 1-3 Providers: Provider visits include in person KIs for Aim 1 (n=28). For Aim 2 providers will be asked to complete a baseline survey (n=98) and KIs (n=28) across the 13 study OPCs. For AIM 3 we will repeat the survey and KIs at 12 months, and separately conduct a sustainability survey at 18 months.

AIM 2 RCT and AIM 4: Patients will be asked to complete a baseline**,** 3-, and 6-month survey. The baseline survey will take place in person. All other surveys will be conducted by telephone, 3 and 6-months. At 6-months follow up patients who report smoking abstinence will be asked to come in person to the OPC and will complete a carbon monoxide test at the OPC to validate self-report.

7.1.4. Methods and Procedures.

AIM 1: Patient Interviews. Experienced interviewers from our partnering institution in Vietnam (i.e., Institute for Social and Medical Studies, ISMS) will conduct these interviews (n=24). The number of interviews will depend on reaching saturation.

AIM 1-3: Health care provider surveys and interviews. All interviews and surveys will be administered by experienced ISMS staff. The interviews will be digitally recorded, transcribed and translated and stored as described above. We will not conduct the interviews if participants refuse to have the interview recorded. Surveys are administered using REDCap on iPads at baseline and 12 months and 18 months post intervention.

AIM 2 RCT and AIM 4: All patient surveys are administered using REDCap. The baseline patient survey is conducted in person at the time of enrollment. All other surveys will be conducted by telephone, 3- and 6- months. At 6-months follow up patients who report smoking abstinence will be asked to come in person to the OPC and will complete a carbon monoxide test at the OPC to validate self-report. The CO test involves the patient blowing through a small cardboard tube into a small handheld monitor which measures the amount of CO expired. A positive test (i.e., still smoking) will be defined as >8 ppm. The CO test visit will take about 20 minutes and the findings will be recorded in REDCap.

***7.1.5. Withdrawal/Early termination visit*.** The clinical site, patients and providers may withdraw from the study at any time.

7.1.6. Unscheduled Visit. For the AIM 2 RCT, unscheduled visits to the nurse counselor will be discouraged. All provider surveys and key informant interviews will be scheduled.
7.1.7. Participant access to study agent at study closure**.** We will not be providing NRT after the study closes.
7.1.8. Prohibited medications**.** There are no prohibited medications. Patients will be able to continue with all of their current medications.
7.1.9. Final Study Visit.
Patients: the visit at 6 months post enrollment is a telephone survey followed by in-person CO confirmation test if the patient reports that they have stopped smoking. We will remind patients about the Quitline service that they can access for additional support and give them the number to call.
Providers: the visit at 18 months post enrollment is an in-person survey to assess sustainability.

8. ASSESSMENT OF SAFETY

8.1 SPECIFICATION OF SAFETY PARAMETERS. This is a minimal risk study. Patients enrolled in the RCT will receive a standard care behavioral intervention from their health care providers (brief counseling plus Quitline counseling) vs provider brief counseling plus the addition of 6 sessions of nurse delivered counseling vs brief physician counseling + multisession nurse delivered counseling +NRT. Eligibility for NRT will be assessed at the time study eligibility is assessed and prior to enrollment. It is important to note that the nicotine gum is an over-the-counter medication, proven safe, with minimal potential side effects. In addition, there are no significant interactions between NRT and HIV treatments (http://www.hivdruginteractions. Patients with a recent history of MI (four weeks), uncontrolled hypertension, arrhythmia, or worsening angina pectoris will not be eligible to enroll. Potential side effects related to NRT will be assessed by the nurse counselor at each counseling visit and reported in the tracking form that counselors use to document visits. The length of treatment with NRT (6 weeks) will generally coincide with those visits. At the time of enrollment participants in the nicotine gum arm will be told of the common side effects (e.g., Rash, mouth, tooth, or jaw problems, palpitations) they may experience and asked to report any problems to the nurse and/or doctor in the clinic or to call the contact number on the consent form. They will also be told how to reduce the likelihood of common side effects, including not smoking while using the gum. Counselor training will include information about potential sides effects and to explore potential reasons for side effects and suggest ways to reduce side effects. The PIs will be contacted within 24 hours if the physician or counselor believes there is an AE or SAE, otherwise, forms will also be reviewed by the ISMS and NYU PIs weekly to assess the need to reduce the dose or discontinue the medication.
8.1.1. Definition of Adverse Events
An adverse event (AE) is any symptom, sign, illness or experience that develops or worsens in severity during the course of the study. Intercurrent illnesses or injuries should be regarded as adverse events. Abnormal results of diagnostic procedures are considered to be adverse events if the abnormality:

- results in study withdrawal
- is associated with a serious adverse event
- is associated with clinical signs or symptoms
- leads to additional treatment or to further diagnostic tests
- is considered by the investigator to be of clinical significance

8.1.2 Definition of Serious Adverse Event
Adverse events are classified as serious or non-serious. A serious adverse event is any AE that is:

- fatal
- life-threatening

- requires or prolongs hospital stay
- results in persistent or significant disability or incapacity
- a congenital anomaly or birth defect
- an important medical event

Important medical events are those that may not be immediately life threatening, but are clearly of major clinical significance. They may jeopardize the subject, and may require intervention to prevent one of the other serious outcomes noted above. For example, drug overdose or abuse, a seizure that did not result in in-patient hospitalization, or intensive treatment of bronchospasm in an emergency department would typically be considered serious. All adverse events that do not meet any of the criteria for serious are regarded as non-serious adverse events.

8.1.3. Definition of Unanticipated Problems

Any incident, experience, or outcome that meets all of the following criteria:

• Unexpected in nature, severity, or frequency (i.e. not described in study-related documents such as the IRB-approved protocol or consent form, the investigators brochure, etc)

• Related or possibly related to participation in the research (i.e. possibly related means there is a reasonable possibility that the incident experience, or outcome may have been caused by the procedures involved in the research)

• Suggests that the research places subjects or others at greater risk of harm (including physical, psychological, economic, or social harm).

8.2. CLASSIFICATION OF AN ADVERSE EVENT

8.2.1. Severity of Event

For AEs not included in the protocol defined grading system, the following guidelines will be used to describe severity.

- **Mild** – Events require minimal or no treatment and do not interfere with the participant's daily activities.
- **Moderate** – Events result in a low level of inconvenience or concern with the therapeutic measures. Moderate events may cause some interference with functioning.
- **Severe** – Events interrupt a participant's usual daily activity and may require systemic drug therapy or other treatment. Severe events are usually potentially life-threatening or incapacitating.

8.2.2. Relationship to the study agent. The PIs will assess if the AE is related or not related to the NRT.

- Related – The AE is known to occur with the study agent, there is a reasonable possibility that the study agent caused the AE, or there is a temporal relationship between the study agent and event. Reasonable possibility means that there is evidence to suggest a causal relationship between the study agent and the AE.
- Not Related – There is not a reasonable possibility that the administration of the study agent caused the event, there is no temporal relationship between the study agent and event onset, or an alternate etiology has been established.

8.2.3. Expectedness. Dr. Shelley and Nguyen will be responsible for determining whether an AE is expected or unexpected. An AE will be considered unexpected if the nature, severity, or frequency of the event is not consistent with the risk information previously described for the study agent.

8.3 TIME PERIOD AND FREQUENCY FOR EVENT ASSESSMENT

The occurrence of an AE or SAE may come to the attention of study personnel during a clinic visit with the provider or the nurse counselor during the intervention period or telephone surveys at 3 months). All AEs, including local and systemic reactions not meeting the criteria for SAEs, will be captured on the appropriate reporting form. Information to be collected includes event description, time of onset, participants' self-assessment of severity, relationship to study product, and time of

resolution/stabilization of the event. All AEs occurring while in the study will be documented appropriately regardless of relationship. All AEs will be followed to adequate resolution.

Any medical condition that is present at the time that the participant is screened will be considered as baseline and not reported as an AE. However, if the study participant's condition deteriorates at any time during the study, it will be recorded as an AE. UPs will be recorded in the data collection system throughout the study.

Changes in the severity of an AE will be documented to allow an assessment of the duration of the event at each level of severity to be performed. AEs characterized as intermittent require documentation of onset and duration of each episode.

The PI will record all reportable events with start dates occurring any time after informed consent is obtained until 7 (for non-serious AEs) or 30 days (for SAEs) after the last day of study participation. At each study visit, the investigator will inquire about the occurrence of AE/SAEs since the last visit. Events will be followed for outcome information until resolution or stabilization. All unresolved adverse events will be followed by the investigator until the events are resolved, the subject is lost to follow-up, or the adverse event is otherwise explained. At the last scheduled visit, the investigator will instruct each subject to report any subsequent event(s) that the subject, or the subject's personal physician, believes might reasonably be related to participation in this study. The investigator will notify the study sponsor of any death or adverse event occurring at any time after a subject has discontinued or terminated study participation that may reasonably be related to this study.

8.4 REPORTING PROCEDURES-NOTIFYING THE IRB AND SPONSOR

8.4.1 Adverse Event Reporting Adverse events will be communicated to the School of Medicine (SoM) and ISMS IRB at the time of continuing review in summary or aggregate form, to the DSMB in aggregate form at each scheduled meeting, and to the Program Officer at NIH in aggregate/summary form in the annual Progress Report. Non-harmful/Expectable events are not recorded or reported to the IRB.

8.4.2 Serious Adverse Event Reporting (1) Report event to IRB within 24 hours using the SoM Report form, (2) Report event to NIH Program Officer and DSMB within 24 hours, (3) Report additional details of event and actions taken to IRB, DSMB, and NIDA Program officer within 72 hours, (4) Communicate DSMB findings to IRB and NIH, (5) Respond to requests for additional information by and/or recommendations from the IRB, DSMB and NIDA.

8.4.3. Unanticipated Problem (UP) Reporting Incidents or events that meet the OHRP criteria for UPs require the creation and completion of an UP-report form. It is the site investigator's responsibility to report UPs to their IRB and to the study sponsor. The UP report will include the following information:

- Protocol identifying information: protocol title and number, PI's name, and the IRB project number;
- A detailed description of the event, incident, experience, or outcome;
- An explanation of the basis for determining that the event, incident, experience, or outcome represents an UP;
- A description of any changes to the protocol or other corrective actions that have been taken or are proposed in response to the UP.

To satisfy the requirement for prompt reporting, UPs will be reported using the following timeline:

- UPs that are SAEs will be reported to the IRB and to DSMB and study sponsor within 24 hours of the investigator becoming aware of the event.

- Any other UP will be reported to the IRB and to DSMB and sponsor within one week of the investigator becoming aware of the problem.
- All UPs will be reported to appropriate institutional officials (as required by an institution's written reporting procedures), the supporting agency head (or designee), and OHRP within 72 hours of the IR's receipt of the report of the problem from the investigator.

8.4.4. Reporting Pregnancy. Pregnant women are excluded from the study because of questions about the safety of NRT during pregnancy.

8.5 REPORTING PROCEDURES NOTIFYING THE STUDY SPONSOR (see section 8.4).

8.6. REPORTING PROCEDURES – NOTIFYING THE FDA (NA)

8.7. HALTING RULES. (NA) This is a minimal risk study. Only those participants randomized to receive the over the counter drug, nicotine gum, will be receiving medication. If patients who are receiving NRT experience side effects these will be addressed as described above. If they must discontinue the nicotine gum they can still choose to continue to participate in the counseling component of the intervention.

8.8. SAFETY OVERSIGHT. It will be the responsibility of the PIs to oversee the safety of the study. This safety monitoring will include careful assessment and appropriate reporting of AEs and SAEs as noted above, as well as the construction and implementation of a site data and safety-monitoring plan. Safety oversight will be under the direction of a DSMB composed of individuals with the appropriate expertise, including a tobacco use treatment expert, an HIV expert and a biostatistician. The DSMB will meet at least semiannually to assess safety and efficacy data on each arm of the study. The DSMB will operate under the rules of an approved charter that will be written and reviewed at the organizational meeting of the DSMB. Each data element that the DSMB needs to assess will be clearly defined. The DSMB will provide its input to Drs. Shelley and Nguyen. The primary responsibilities of the DSMB are to 1) periodically review and evaluate the accumulated study data for participant safety, study conduct and progress, and, when appropriate, efficacy, and 2) make recommendations concerning the continuation, modification, or termination of the trial. The DSMB will consider study-specific data as well as relevant background knowledge about the intervention, or patient population under study.

9.0 STATISTICAL CONSIDERATIONS

9.1 STATISTICAL AND ANALYTIC PLANS AND METHODS (SAP)

AIM 1: To explore barriers to tobacco cessation among HIV+ smokers.

Survey data will be summarized using descriptive statistics. Qualitative data from the audio recordings of the KIs will be transcribed verbatim in Vietnamese by ISMS, translated into English and back translated. Two trained NYU and ISMS coders will analyze the interview data using a priori concepts that emerge from the quantitative data and both an inductive and deductive approach (open coding and coding of theoretical constructs). We will use Atlas ti. to facilitate our multi-step iterative coding and analyses. Data coding will begin with independent reading of the transcripts to identify preliminary themes and relevant patterns, followed by focused coding to identify clustered concepts and to organize ideas, and finally identification of major themes. Throughout, coders will meet to review their coding, conduct team debriefing meetings, and reach consensus on code names and meanings. Once all transcripts have been collaboratively coded, analytic domains will be identified and major and minor thematic areas described.

AIM 2: (Primary effectiveness endpoint analysis) To compare the effectiveness of three interventions to increase tobacco cessation among PLHW who smoke. The primary analytic approach used to compare study arms is conditional logistic regression.[17,18] This fixed effects approach was chosen as primary because of the modest number of sites and the fact that they are not very heterogeneous. Because some methodologists have advocated mixed effects models even when the number of sites is small,[19,20] we will use a multilevel logistic regression model as an alternative, sensitivity analysis approach. In an intent-to-treat analysis of all study entrants,

conditional logistic regression analysis will compare study arms on the primary outcome, 7-day point prevalence CO confirmed (<8 ppm) tobacco abstinence captured at 6-months follow up. The outcome will be regressed on indicator variables for study arm and OPC site will be the stratification variable. Pairwise comparisons of arms will be undertaken to determine which arms are significantly different. Pairwise comparisons will be adjusted for multiple comparisons using the Tukey method.[21] Effect magnitudes will be based on odds ratios for the pairwise comparisons, which will be calculated by exponentiating the coefficients for pairwise contrasts. Secondary cessation outcomes, HIV-related outcomes, and hypothesized mediators (e.g., risk perceptions, self-efficacy) also will be analyzed with conditional logistic regression, or a different fixed effects regression appropriate to the type of outcome variable. Multilevel regression will be used as sensitivity analysis. To calculate indirect effects of study arm on abstinence via potential mediators, the Mplus statistical modeling software will be used with bootstrapping of products of direct effects.[22,23] Patient characteristics (e.g., sex, substance use, health) will be included as covariates in all analysis and explored as potential effect modifiers. To examine effects of fidelity or "dose" of intervention, we will estimate effects of receiving the full dose of each intervention component, or not, using *complier average causal effect* estimation.[24,25] Using patient characteristics associated with receiving a full dose among those offered each component, the Complier Average Casual Effect (CACE) approach finds similar patients not offered the component but likely to receive a full dose if they had been offered. This permits patients who receive the full dose of specific components to be compared with similar patients not offered the component, in order to estimate intervention effects for "compliers" only. Identifying the intervention's impact on compliers deepens our understanding of to whom the results apply, and to whom they do not, thereby suggesting possible adaptations of components with less than perfect fidelity or uptake. CACE is a type of per protocol analysis, with similarities to other approaches for estimating causal effects with noncompliance or partial dose, which constitutes a shift from estimating effects of treatment *offered* to treatment *received.*

AIM 3: Given the small sample size of provider surveys, we will use descriptive statistics to summarize the quantitative data and assess changes pre and post-trial in provider and clinic-level factors. We will use bivariate analysis to explore differences by study site and to assess relationships between provider and perceived intervention characteristics (Table 3) and adherence (fidelity) to standard care (i.e., Ask, Advise, Assist, Refer) based on their self-report (survey Appendix). Qualitative data from providers and program directors will be transcribed, translated, and back translated. Dr. Nguyen's fluency in English and Vietnamese will ensure "conceptual equivalence." Two trained NYU and ISMS coders will analyze the interview data using a priori concepts that emerge from the quantitative data and both an inductive and deductive approach (open coding and coding of theoretical constructs).[26,27] We will use Atlas ti. to facilitate our multi-step iterative coding and analyses. Data coding will begin with independent reading of the transcripts to identify preliminary themes and relevant patterns, followed by focused coding to identify clustered concepts and to organize ideas, and finally identification of major themes. Throughout, coders will meet to review their coding, conduct team debriefing meetings, and reach consensus on code names and meanings. Once all transcripts have been collaboratively coded, analytic domains will be identified and major and minor thematic areas described.

Our analysis will also integrate the data to examine how qualitative themes converge with or expand on quantitative findings. This will include creating a table that examines key constructs from our frameworks with responses from all sources of data. We will then triangulate findings from pre and post assessments by developing a thematic matrix that includes system, clinic and provider-level characteristics, and compare side by side, those factors that were identified as faciliating or hindering the implementation process. Findings will be used to create a heuristic model to inform further optimization of the implementation process, and sustainability, if found

effective. Data will be shared with the SAC during meetings to inform discussions about strategies for faciliating sustainablity and dissemination.

AIM 4: When comparing smokers and non-smokers at baseline on the HFIAS, power is 80% to detect a standardized mean difference of d=0.46 (about half a standard deviation, a difference of 3 HFIAS points assuming a standard deviation of 6) given the proposed sample size of n= 150 (75 smokers and 75 non-smokers). When estimating the relation between income and HFIAS, power is 80% to detect a squared multiple correlation of R^2=.05 given the proposed sample size of n=150. When estimating the impact of smoking cessation on HFIAS at the 6-month follow-up, power is 80% to detect a standardized mean difference of d=0.89 (about nine tenths of a standard deviation, a difference of 6 HFIAS points) given the proposed sample size of n=75 smokers at baseline and assuming at least 16% of smokers (n=12) achieve cessation.

9.2 STATISTICAL HYPOTHESIS. The primary hypothesis is that more intensive counseling delivered by a trained nurse (ARM 2) will be more effective than provider brief counseling plus Quitline counseling (ARM 1) in increasing smoking abstinence. We further hypothesize that adding nicotine replacement therapy to the more intensive counseling will increase cessation rates more than the intensive counseling alone (ARM 3).

9.3 PLANNED INTERIM ANALYSIS

We will conduct descriptive statistics to be presented to the DSMB at the biannual meetings. This will include analysis of recruitment progress to address any barriers and to review the safety data.

10. SOURCE DOCUMENTS AND ACCESS TO SOURCE DATA/DOCUMENTS. The study case report form (CRF) is the primary data collection instrument for the study. All data requested on the CRF must be recorded. All missing data must be explained. If a space on the CRF is left blank because the procedure was not done or the question was not asked, write “N/D”. If the item is not applicable to the individual case, write “N/A”. All entries should be printed legibly in black ink. If any entry error has been made, to correct such an error, draw a single straight line through the incorrect entry and enter the correct data above it. All such changes must be initialed and dated. For clarification of illegible or uncertain entries, print the clarification above the item, then initial and date it. Access to study records will be limited to IRB-approved members of the study team. The investigator will permit study-related monitoring, audits, and inspections by the IRB, the sponsor, government regulatory bodies, and University compliance and quality assurance groups of all study related documents (e.g. source documents, regulatory documents, data collection instruments, study data etc.). The investigator will ensure the capability for inspections of applicable study-related facilities. Participation as an investigator in this study implies acceptance of potential inspection by government regulatory authorities and applicable University compliance and quality assurance offices.

11. QUALITY ASSURANCE AND QUALITY CONTROL (QC).

QC procedures will be implemented beginning with the data entry system and data QC checks that will be run on the database will be generated. Any missing data or data anomalies will be communicated to the site(s) for clarification/resolution. Following written Standar Operating Procedures, the monitors will verify that the clinical trial is conducted and data are generated, documented (recorded), and reported in compliance with the protocol, GCP, and the applicable regulatory requirements (e.g., Good Laboratory Practices (GLP), Good Manufacturing Practices (GMP)). The investigational site will provide direct access to all trial related sites, source data/documents, and reports for the purpose of monitoring and auditing by the sponsor, and inspection by local and regulatory authorities.

12. ETHICS/PROTECTION OF HUMAN SUBJECTS

The investigator will ensure that this study is conducted incompliance with the Declaration of Helsinki, Council for International Organizations of Medical Science (CIOMS), International Ethical Guidelines for Biomedical Research Involving Human Subjects (2002), or another country's ethical policy statement, whichever provides the most protection to human subjects.

12.1 INFORMED CONSENT PROCESS AIM 1. *Individual Interview with patients documentation of consent*: Patients will be screened by the nurse for eligibility as described above, asked to participate if they meet eligibility criteria, and given a date and time to speak to the RA. Verbal consent will be obtained prior to completing the screening questionnaire. All study related activities and study data collection will occur only AFTER a consent is obtained. We are requiring that interviews are audiotaped and therefore will let potential participants know that at the time they are screened to offer them an opportunity to decline at that time. We will again explain that interviews will be audio taped prior to beginning the interview at which time they will have another chance to opt out. We have requested a waiver of written consent and authorization. Participants will be given an information sheet that includes a number to call if they have additional questions. The interviews will take about 45-60 minutes. We will make it clear to the subjects that participation in the interviews is separate from any ongoing care they may receive in at the clinic and that participation is voluntary. We will emphasize that they are free to stop participation at any time and are free to refuse to answer specific questions in any assessment and can decline participation in any intervention activity.

Key informant interview with provider verbal consent: For the preintervention provider KIs we will obtain verbal consent at the time of the interview. We are requiring that interviews are audiotaped and therefore will let potential participants know that the time they are screened to offer them an opportunity to decline at that time. We have requested a waiver of written consent and authorization.

Pilot testing of RCT: The same process used for Aim 2 will be used to conduct screening and consenting of patients for the pilot test of ARM 1 and 3 in one OPC.

AIM 2 and AIM 3. *Provider survey and KIs documentation of consent*: Health care workers will be told about the study at a group meeting. It will be made clear to them that participation is voluntarily and they don't have to participate. This will be repeated at the time of the consent process. All health workers are eligible. Research staff will follow-up to contact providers and staff to make appointments for interviews and to administer the baseline and follow up surveys. Written consent will be obtained at the time of the baseline survey and interview prior to starting any data collection. Health care providers who participate in interviews and surveys will be asked to complete the survey and KIs again at 12 months post intervention. None of the research staff have any role in evaluating the employees or role in firing or hiring decisions. We will obtain written consent because of the longitudinal nature of the data collection (i.e., providers will be asked to participate in a survey at baseline and then at 12 months, in KIs at baseline and 12 months, and a sustainability survey at 18 months). Health care workers have the right to refuse to participate without any compromise to their employment or status.

AIM 2 RCT. *Patient survey.* A nurse who typically registers patients when they arrive at the OPC will screen all patients for tobacco use as part of that registration process. If they are a smoker the nurse will briefly describe the study and explain that those patients who are interested in participating will be screened for eligibility. Those patients who meet eligibility criteria, based on this initial screening will be introduced to the RA to complete the consent and baseline assessment procedures before their provider visit.

AIM 4. *Patient survey for non-smokers.* The consent process will follow the same steps as AIM 2 RCT above.

For all participants, we will emphasize that they are free to stop participation at any time and are free to refuse to answer specific questions in any assessment and can decline participation in any intervention activity. Participants will be informed that their decision to participate or decline to enroll in the study or any aspect of the study will not affect any services they receive at the clinic or their employment. Patients who smoke and decline to participate, will be offered the educational materials which include the Quitline number.

12.2 PARTICIPANT AND DATA CONFIDENTIALITY

Only the (1) Participant Log, (2) computerized tracking database, and (3) paper consent form will link the participant's name to his/her identification number. The Participant Log is password-protected electronic file containing a list of identification numbers, the participant's name, intervention arm assignments (0, 1, 2), and date of screening for and enrollment into the study. A secure, online, password-protected database built on a REDCap platform and linked to a secure web-based platform will be used to manage recruitment, eligibility assessment, randomization to study conditions, and survey collection and tracking. If paper copies of the patient eligibility screener are collected, the nurse will keep these in a locked cabinet at each OPC. Paper copies of the screener and consent forms will be picked up weekly by the ISMS RA and transferred to a locked cabinet at ISMS office, where only the RA and PI in Vietnam will have access to the cabinets. Paper copies of the screener will be coded and entered into REDCap and destroyed once entered into the database. Information about study subjects will be kept confidential and managed according to the requirements of the Health Insurance Portability and Accountability Act of 1996 (HIPAA). Those regulations require a signed subject authorization informing the subject of the following:

- What protected health information (PHI) will be collected from subjects in this study
- Who will have access to that information and why
- Who will use or disclose that information
- The rights of a research subject to revoke their authorization for use of their PHI.

In the event that a subject revokes authorization to collect or use PHI, the investigator, by regulation, retains the ability to use all information collected prior to the revocation of subject authorization. For subjects that have revoked authorization to collect or use PHI, attempts should be made to obtain permission to collect at least vital status (i.e. that the subject is alive) at the end of their scheduled study period.

13. DATA HANDLING AND RECORD KEEPING

13.1 DATA COLLECTION AND MANAGEMENT RESPONSIBILITIES. We will use REDCap to collect survey data for providers and staff. Paper versions of the patient eligibility screener and signed consent forms will be stored in locked cabinets. Qualitative interviews will be digitally recorded. The RAs will download the recordings to a password protected computer on the same day the data is collected. The recordings will be transcribed by ISMS staff who did not attend the interviews within a week of recording and any identifiable information will be removed. The audio recordings will not have any identifiable information. Once transcribed, the taped session will be deleted by the RA. Data will be sent to the Quitline by ISMS via email using encrypted and password protected documents. Passwords to access the documents will be sent in a separate email. The Quitline will keep the data in a secure server at Bach Mai Hospital. The NYU project analyst will conduct all database development, data management and data quality assurance activities for the study under the supervision of the study biostatistician.

13.2 STUDY RECORDS RETENTION Study documents will be retained for at least 5 years after final reporting/publication. No records will be destroyed without the written consent of the sponsor.

13.3 PROTOCOL DEVIATIONS All deviations in protocol will be addressed in study source documents, reported to the NYULMC IRB.

13.4 PUBLICATION AND DATA SHARING POLICY

This study will comply with the NIH Public Access Policy, which ensures that the public has access to the published results of NIH funded research. It requires scientists to submit final peer-reviewed journal manuscripts that arise from NIH funds to the digital archive PubMed Central upon acceptance for publication.

14. STUDY FINANCES

14.1 FUNDING SOURCE. This study is financed through a grant from the US National Cancer Institute.

14.2 COSTS TO PARTICIPANTS. No costs will incur as a result of participating in the study.

14.3 PARTICIPANT REIMBURSEMENTS OR PAYMENTS. The following is a list of the payments developed by our partners in Viet Nam: 1) Quantitative survey with smokers: $3/person/each time (4 surveys per subject, at baseline, 3 and 6 months), 2) Health care provider surveys: $5/person/each time (baseline survey and 12 months post intervention survey). 3) Interviews with smokers: $4/person. 4) Key informant interviews: $5 per person. 5) sustainability survey: $4.5 per person. Our team has extensive experience enrolling participants in research in Viet Nam and therefore we believe we have developed an incentive plan that is consistent with the norms in Viet Nam and will reduce the risk of coercion.

15. STUDY ADMINISTRATION

15.1 STUDY LEADERSHIP

This protocol describes a multiple-PI collaborative project bringing together two well-established investigators with complementary skills and expertise, a shared commitment to the objectives of this study and a history of collaboration. A MPI plan will address the need for leadership with multidisciplinary expertise. The MPIs include Dr. Donna Shelley a Professor in the Dept of Population Health at the NYU School of Medicine and Professor of Public Health Policy and Management in the NYU School of Public Health. Dr. Nam Nguyen is Director of ISMS. The partnership between Drs. Shelley and Nguyen bridges expertise in epidemiology, policy evaluation, behavioral science, implementation science and health services research. Both investigators have established a track record for collaborative research, which provides evidence that they are capable of acting at an appropriate level of authority and responsibility to direct the project.
We will also convene an external advisory committee to ensure that the research is aligned with key stakeholders' goals for understanding the impact of the policy, and to provide a platform for disseminating findings and scale up of best practices more widely. Members will include leaders from the Ministry of Health, academic institutions, and HIV clinical leadership at the Provincial and District level. Members will meet twice a year with the study investigators. We will engage them to review assessment tools, provide national and local context.

16. CONFLICT OF INTEREST POLICY. The independence of this study from any actual or perceived influence is critical. Therefore, any actual conflict of interest of persons who have a role in the design, conduct, analysis, publication, or any aspect of this study will be disclosed and managed. Furthermore, persons who have a perceived conflict of interest will be required to have such conflicts managed in a way that is appropriate to their participation in the study. The study leadership in conjunction with the NIH National Cancer Institute – Tobacco Control Research Branch has established policies and procedures for all study group members to disclose all conflicts of interest and will establish a mechanism for the management of all reported dualities of interest.

17. REFERENCES

5. Van Minh H, Giang KB, Ngoc NB, Hai PT, Huyen DT, Khue LN, Lam NT, Nga PT[5], Quan NT, Xuyen NT. Prevalence of tobacco smoking in Vietnam: findings from the Global Adult Tobacco Survey 2015. Int J Public Health. 2017 Feb;62(Suppl 1):121-129. doi: 10.1007/s00038-017-0955-8.
6. Fiore M, United States. Tobacco Use and Dependence Guideline Panel. Treating tobacco use and dependence: 2008 update. 2008 update ed. [Rockville, Md.]: U.S. Dept. of Health and Human Services, Public Health Service; 2008
7. Stead LF, Lancaster T. Combined pharmacotherapy and behavioral interventions for smoking cessation. Cochrane Database Syst Rev. 2016 Mar 24;3:CD008286. doi: 10.1002/14651858.CD008286.pub3.
8. Pool ER, Dogar O, Lindsay RP, Weatherburn P, Siddiqi K. Interventions for tobacco use cessation in people living with HIV and AIDS. Cochrane Database Syst Rev. 2016 Jun 13;(6):CD011120.
9. Niaura R[1], Chander G, Hutton H, Stanton C. Interventions to address chronic disease and HIV: strategies to promote smoking cessation among HIV-infected individuals. Curr HIV/AIDS Rep. 2012 Dec;9(4):375-84
10. Harris JK. Connecting discovery and delivery: the need for more evidence on effective smoking cessation strategies for people living with HIV/AIDS. Am J Pub Health 2010;100(7):1245-1249.
11. Keith A, Dong Y, Shuter J, Himelhoch S. Behavioral Interventions for Tobacco Use in HIV-Infected Smokers: A Meta-Analysis. J Acquir Immune Defic Syndr. 2016 Aug 15;72(5):527-33.
12. Gritz ER, Danysh HE et al. Long term outcomes of a cell phone-delivered intervention for smokers living with HIV/AIDS. Clin Infec Dis. 2013;57(4):608-15.
13. Lancaster T, Stead LF.Individual behavioural counselling for smoking cessation. Cochrane Database Syst Rev. 2017;31;3:CD001292. doi: 10.1002/14651858.CD001292
14. Fiore M, United States. Tobacco Use and Dependence Guideline Panel. Treating tobacco use and dependence: 2008 update. 2008 update ed. [Rockville, Md.]: U.S. Dept. of Health and Human Services, Public Health Service; 2008
15. Stead LF, Lancaster T. Combined pharmacotherapy and behavioral interventions for smoking cessation. Cochrane Database Syst Rev. 2016 Mar 24;3:CD008286. doi: 10.1002/14651858.CD008286.pub3.
16. West R, Raw M, McNeill A, Stead L, Aveyard P, Bitton J, Stapleton J, McRobbie H, Pokhrel S, Lester-George A, Borland R. Health-care interventions to promote and assist tobacco cessation: a review of efficacy, effectiveness and affordability for use in national guideline development. Addiction. 2015 Sep;110(9):1388-403.
17. Sorensen G, Barbeau E, Hunt MK, Emmons K. Reducing social disparities in tobacco use: a social-contextual model for reducing tobacco use among blue-collar workers. Am J Public Health. 2004 Feb;94(2):230-9.
18. Miller WR, Rollnick S. Motivational Interviewing: Preparing people to change addictive behaviors. New York: Guilford Press; 1991.
19. Marlatt GA. Relapse prevention: Maintenance strategies in the treatment of addictive behaviors: TheGuilford Press; 2005
20. Strengthening health systems for treating tobacco use: building capcity for tobacco control. WHO Tobacco Control Package. http://www.who.int/tobacco/publications/building_capacity/training_package/treatingtobaccodependence/en/. Accessed 6-19-19

21. Moyers T, Manuel J, Ernst D. Motivational interviewing treatment integrity coding manual 4.2.1. 2014.Unpublished manual. [cited 2015 September 23]. Available from: http://casaa.unm.edu/download/MITI4_2.pdf
22. Hosmer, D. W. and Lemeshow, S. 2005. Logistic Regression, Conditional. Encyclopedia of Biostatistics.
23. Peduzzi P, Henderson W, Hartigan P, Lavori, P. (2002). Analysis of randomized controlled trials. Epidemiologic Reviews, 24, 26-38
24. Senn, S. (1998). Some controversies in planning and analyzing multi-centre trials. Statistics in Medicine, 17, 1753-1765.
25. Moerbeek M, van Breukelen GJP, Berger MPF. A comparison between traditional methods and multilevel regression for the analysis of multicenter intervention studies. J Clin Epi. 2003; 56, 341-350.
26. Bretz F, Hothorn, T., & Westfall, P. H. (2011). *Multiple comparisons using R*. Boca Raton: CRC Press.
27. Allison, P.D. (2009). *Fixed effects regression models* (Vol. 160). SAGE publications.
28. Muthén LK, Muthén BO. (998-2017). *Mplus user's guide, Eighth Edition*. Los Angeles, CA.
29. MacKinnon DP, Lockwood CM, Hoffman, J.M., West, S.G., & Sheets, V. (2002). A comparison of methods to test mediation and other intervening variable effects. *Psychological Methods, 7*, 83-104.
30. Creswell JW. Qualitative inquiry and research design: Choosing among five traditions. Thousand Oaks,CA: SAGE; 1998.
31. Davies D, Dodd J. Qualitative research and the question of rigor. Qual Health Res 2002;12:279-289
